# Brain. Conscious and Unconscious Mechanisms of Cognition, Emotions, and Language

**DOI:** 10.3390/brainsci2040790

**Published:** 2012-12-18

**Authors:** Leonid Perlovsky, Roman Ilin

**Affiliations:** 1 Athinoula A. Martinos Center for Biomedical Imaging, Harvard University, Charlestown, MA 02129, USA; 2 Air Force Research Laboratory, Wright-Patterson AFB, OH 45433, USA; E-Mail: roman.ilin@hanscom.af.mil

**Keywords:** conscious, unconscious, cognition, emotions, language, knowledge instinct, dynamic logic, grounded symbols, dual hierarchy, bottom-up signals, top-down signals, cognitive dissonances, beautiful, sublime, music

## Abstract

Conscious and unconscious brain mechanisms, including cognition, emotions and language are considered in this review. The fundamental mechanisms of cognition include interactions between bottom-up and top-down signals. The modeling of these interactions since the 1960s is briefly reviewed, analyzing the ubiquitous difficulty: incomputable combinatorial complexity (CC). Fundamental reasons for CC are related to the Gödel’s difficulties of logic, a most fundamental mathematical result of the 20th century. Many scientists still “believed” in logic because, as the review discusses, logic is related to consciousness; non-logical processes in the brain are unconscious. CC difficulty is overcome in the brain by processes “from vague-unconscious to crisp-conscious” (representations, plans, models, concepts). These processes are modeled by dynamic logic, evolving from vague and unconscious representations toward crisp and conscious thoughts. We discuss experimental proofs and relate dynamic logic to simulators of the perceptual symbol system. “From vague to crisp” explains interactions between cognition and language. Language is mostly conscious, whereas cognition is only rarely so; this clarifies much about the mind that might seem mysterious. All of the above involve emotions of a special kind, aesthetic emotions related to knowledge and to cognitive dissonances. Cognition-language-emotional mechanisms operate throughout the hierarchy of the mind and create all higher mental abilities. The review discusses cognitive functions of the beautiful, sublime, music.

## 1. Overcoming Past Mathematical Difficulties

According to modern neuroscience, object perception involves bottom-up signals from sensory organs and top-down signals from internal mind’s representations (memories) of objects. During perception, the mind matches subsets of bottom-up signals corresponding to objects with representations of object in the mind (and top-down signals). This produces object recognition; it activates brain signals leading to mental and behavioral responses [1,2,3,4,5]. This section briefly summarizes mathematical development in artificial intelligence, pattern recognition, and other computational methods used in cognitive science for modeling brain-mind processes. We discuss the fundamental difficulties preventing mathematical modeling of perception, cognition, emotions, and the role of dynamic logic (DL) in overcoming these difficulties.

### 1.1. Computational Complexity since the 1950s

Developing mathematical descriptions of the very first recognition step in this seemingly simple association-recognition-understanding process has not been easy, a number of difficulties have been encountered during the past 50 years. These difficulties were summarized under the notion of combinatorial complexity (CC) [6]. CC refers to multiple combinations of bottom-up and top-down signals, or more generally to combinations of various elements in a complex system; for example, recognition of a scene often requires concurrent recognition of its multiple elements that could be encountered in various combinations. CC is computationally prohibitive because the number of combinations is very large: for example, consider 100 elements (not too large a number); the number of combinations of 100 elements is 100^100^, exceeding the number of all elementary particle events in the life of the Universe; no computer would ever be able to compute that many combinations. Although, the story might sound “old”, we concentrate here on those aspects of mathematical modeling of the brain-mind, which remain current and affect thinking in computational modeling and in cognitive science of many scientists today.

The problem of CC was first identified in pattern recognition and classification research in the 1960s and was named “the curse of dimensionality” [7]. It seemed that adaptive self-learning algorithms and neural networks could learn solutions to any problem “on their own”, if provided with a sufficient number of training examples. The following decades of developing adaptive statistical pattern recognition and neural network algorithms led to a conclusion that the required number of training examples often was combinatorially large. This remains true about recent generation of algorithms and neural networks, which are much more powerful than those in the 1950s and 60s. Training had to include not only every object in its multiple variations, angles, *etc.*, but also combinations of objects. Thus, self-learning approaches encountered CC of learning requirements. 

Rule systems were proposed in the 1970s to solve the problem of learning complexity [8,9]. Minsky suggested that learning was a premature step in artificial intelligence; Newton “learned” Newtonian laws, most of scientists read them in the books. Therefore, Minsky has suggested, knowledge ought to be input in computers “ready made” for all situations and artificial intelligence would apply these known rules. Rules would capture the required knowledge and eliminate a need for learning. Chomsky’s original ideas concerning mechanisms of language grammar related to deep structure [10] were also based on logical rules. Rule systems work well when all aspects of the problem can be predetermined. However, in the presence of variability, the number of rules grew; rules became contingent on other rules and combinations of rules had to be considered. The rule systems encountered CC of rules. 

In the 1980s, model systems were proposed to combine advantages of learning and rules-models by using adaptive models [11,12,13,14,15,16,17,18]. Existing knowledge was to be encapsulated in models and unknown aspects of concrete situations were to be described by adaptive parameters. Along similar lines went the principles and parameters idea of Chomsky [19]. Fitting models to data (top-down to bottom-up signals) required selecting data subsets corresponding to various models. The number of subsets, however, is combinatorially large. A general popular algorithm for fitting models to the data, multiple hypotheses testing [20] is known to face CC of computations. Model-based approaches encountered computational CC (N and NP complete algorithms). None of the past computational approaches modeled specifically human, “aesthetic emotions” (discussed later) related to knowledge, cognitive dissonances, beautiful, and “higher” cognitive abilities.

### 1.2. Logic, CC, and Amodal Symbols

Amodal symbols and perceptual symbols described by perceptual symbol system (PSS) [21] differ not only in their representations in the brain, but also in their properties that are mathematically modeled in the referenced papers. This mathematically fundamental difference and its relations to CC of matching bottom-up and top-down signals are the subjects of this section. (A specific reason for connecting our cognitive-mathematical analysis to PSS is that it is a well recognized cognitive theory, giving a detailed non-mathematical description of many cognitive processes; later we discuss that PSS is incomplete and mathematically untenable for abstract concepts, language-cognition interaction, and for aesthetic emotions; necessary modifications are described in the following references [22,23,24].)

The fundamental reasons for CC are related to the use of formal logic by algorithms and neural networks [6,25,26]. Logic serves as a foundation for many approaches to cognition and linguistics; it underlies most of computational algorithms. But its influence extends far beyond, affecting cognitive scientists, psychologists, and linguists, who do not use complex mathematical algorithms for modeling the mind. All of us operate under the influence of formal logic, which roots are more than 2000 years old, making a more or less conscious assumption that the mechanisms of logic serve as the basis of human cognition. As discussed in details later, our minds are unconscious about its illogical foundations. We are mostly conscious about a small part of the mind mechanisms, which is approximately logical. Our intuitions, therefore, are unconsciously affected by the bias toward logic. Even when the laboratory data drive thinking away from logical mechanisms, humans have difficulties overcoming the logical bias [1,4,25,27,28,29,30,31,32,33,34].

The relationships between logic, cognition, and language have been a source of longstanding controversy. The widely accepted story is that Aristotle founded logic as a fundamental mind mechanism, and only during the recent decades science overcame this influence. We would like to emphasize the opposite side of this story. Aristotle assumed a close relationship between logic and language. He emphasized that logical statements should not be formulated too strictly and language inherently contains the necessary degree of precision. According to Aristotle, logic serves to communicate already made decisions [32]. The mechanism of the mind relating language, cognition, and the world Aristotle described as forms. Today we call similar mechanisms mental representations, or concepts, or simulators in the mind. Aristotelian forms are similar to Plato’s ideas with a marked distinction, forms are dynamic: their initial states, before learning, are different from their final states of concepts [35]. Aristotle emphasized that initial states of forms, forms-as-potentialities, are not logical (*i.e.*, vague), but their final forms, forms-as-actualities, attained in the result of learning, are logical. This fundamental idea was lost during millennia of philosophical arguments. As discussed below, this Aristotelian process of dynamic forms corresponds to the mathematical model, DL, for processes of perception and cognition, and to Barsalou idea of PSS simulators.

The founders of formal logic emphasized a contradiction between logic and language. In the 19th century George Boole and the great logicians following him, including Gottlob Frege, Georg Cantor, David Hilbert, and Bertrand Russell (see [36] and references therein) eliminated the uncertainty of language from mathematics, and founded formal mathematical logic, the foundation of the current classical logic. Hilbert developed an approach named formalism, which rejected intuition as a matter of scientific investigation and formally defined scientific objects in terms of axioms or rules. In 1900 he formulated famous Entscheidungsproblem: to define a set of logical rules sufficient to prove all past and future mathematical theorems. This was a part of “Hilbert’s program”, which entailed formalization of the entire human thinking and language. Formal logic ignored the dynamic nature of Aristotelian forms and rejected the uncertainty of language. Hilbert was sure that his logical theory described mechanisms of the mind. “The fundamental idea of my proof theory is none other than to describe the activity of our understanding, to make a protocol of the rules according to which our thinking actually proceeds.” [37]. However, Hilbert’s vision of formalism explaining mysteries of the human mind came to an end in the 1930s, when Gödel [38] proved internal inconsistency of formal logic. This development called Gödel theory is considered among most fundamental mathematical results of the previous century. Logic, that was believed to be a sure way to derive truths, turned out to be basically flawed. This is a reason why theories of cognition and language based on formal logic are inherently flawed. 

There is a close relation between logic and CC. It turned out that combinatorial complexity of algorithms is a finite-system manifestation of the Gödel’s theory [30]. If Gödelian theory is applied to finite systems (all practically used or discussed systems, such as computers and brain-mind, are finite), CC is the result, instead of the fundamental inconsistency. Algorithms matching bottom-up and top-down signals based on formal logic have to evaluate every variation in signals and their combinations as separate logical statements. A large, practically infinite number of combinations of these variations cause CC. 

This general statement manifests in various types of algorithms in different ways. Rule systems are logical in a straightforward way, and the number of rules grows combinatorially. Pattern recognition algorithms and neural networks are related to logic in learning procedures: every training sample is treated as a logical statement (“this is a chair”) resulting in CC of learning. Multivalued logic and fuzzy logic were proposed to overcome limitations related to logic [39]. Yet the mathematics of multivalued logic is no different in principle from formal logic [31]. Fuzzy logic uses logic to set a degree of fuzziness. Correspondingly, it encounters a difficulty related to the degree of fuzziness: if too much fuzziness is specified, the solution does not achieve a needed accuracy, and if too little, it becomes similar to formal logic. If logic is used to find the appropriate fuzziness for every model at every processing step, then the result is CC. The mind has to make concrete decisions, for example one either enters a room or does not; this requires a computational procedure to move from a fuzzy state to a concrete one. But fuzzy logic does not have a formal procedure for this purpose; fuzzy systems treat this decision on an ad-hoc logical basis. A more general summary of this analysis relates CC to logic in the process of learning. Learning is treated in all past algorithms and in many psychological theories as involving learning from examples. An example such as “this is a chair” is a logical statement. Hence the ubiquitous role of logic and CC. 

Is logic still possible after Gödel’s proof of its incompleteness? The contemporary state of this field was reviewed in [26]. It appears that logic after Gödel is much more complicated and much less logical than was assumed by founders of artificial intelligence. CC cannot be solved within logic. Penrose thought that Gödel’s results entail incomputability of the mind processes and testify for a need for new physics “correct quantum gravitation”, which would resolve difficulties in logic and physics [40]. An opposite position in [25,30,31] is that incomputability of logic does not entail incomputability of the mind. These references add mathematical arguments to Aristotelian view that logic is not the basic mechanism of the mind.

To summarize, various manifestations of CC are all related to formal logic and Gödel theory. Rule systems rely on formal logic in a most direct way. Even mathematical approaches specifically designed to counter limitations of logic, such as fuzzy logic and the second wave of neural networks (developed after the 1980s) rely on logic at some algorithmic steps. Self-learning algorithms and neural networks rely on logic in their training or learning procedures: Every training example is treated as a separate logical statement. Fuzzy logic systems rely on logic for setting degrees of fuzziness. CC of mathematical approaches to the mind is related to the fundamental inconsistency of logic. All past algorithms and theories capable of learning involved logic in their learning procedures. Therefore logical inspirations, leading early cognitive scientists to amodal brain mechanisms, could not realize their hopes for mathematical models of the brain-mind.

Why did the outstanding mathematicians of the 19th and early 20th century believe in logic to be the foundation of the mind? Even more surprising is the belief in logic after Gödel. Gödelian theory was long recognized among most fundamental mathematical results of the 20th century. How is it possible that outstanding minds, including founders of artificial intelligence, and many cognitive scientists and philosophers of mind insisted that logic and amodal symbols implementing logic in the mind are adequate and sufficient? The answer, in our opinion, might be in the “conscious bias”. As we discuss, non-logical operations making up more than 99.9% of the mind functioning are not accessible to consciousness [4,25,27,29,30]. However, our consciousness functions in a way that makes us unaware of this. In subjective consciousness we usually experience perception and cognition as logical. Our intuitions are “consciously biased”. This is why amodal logical symbols, which describe a tiny fraction of the mind mechanisms, have seemed to many the foundation of the mind [4,25,28,29,30,31,32,33,34,41].

Another aspect of logic is that it lacks dynamics; logic operates with static statements such as “this is a chair”. Classical logic is good at modeling structured statements and relations, yet it misses the dynamics of the mind and faces CC, when attempts to match bottom-up and top-down signals. The essentially dynamic nature of the brain-mind is not represented in mathematical foundations of logic. Dynamic logic discussed in the next section is a logic-process. It overcomes CC by automatically choosing the appropriate degree of fuzziness-vagueness for every mind’s concept at every moment. DL combines advantages of logical structure and connectionist dynamics. This dynamics mathematically represents the learning process of Aristotelian forms (which are opposite to classical logic as mentioned) and serves as a foundation for PSS concepts and simulators. 

### 1.3. Dynamic Logic-Process

DL models perception as an interaction between bottom-up and top-down signals [25,30,31,32,33,34]. This section concentrates on the basic relationship between the brain processes and the mathematics of DL. To concentrate on this relationship, we much simplify the discussion of the brain structures. We discuss visual recognition of objects as if the retina and the visual cortex each consist of a single processing level of neurons where recognition occurs (which is not true, detailed relationship of the DL process to brain is considered in given references). Perception consists of the association-matching of bottom-up and top-down signals. Sources of top-down signals are mental representations, memories of objects created by previous simulators [21]; these representations model the patterns in bottom-up signals. In this way they are concepts (of objects), symbols of a higher order than bottom-up signals; we call them concepts or mental models. In perception processes the models are modified by learning and new models are formed; since an object is never encountered exactly the same as previously, perception and cognition are always learning processes. The DL processes along with concept-representations are mathematical models of the PSS simulators. The bottom-up signals, in this simplified discussion, are a field of neuronal synapse activations in visual cortex. Sources of top-down signals are mental representation-concepts or, equivalently, model-simulators (for short, models; please notice this dual use of the word model, we use “models” for mental representation-simulators, which match-model patterns in bottom-up signals; and we use “models” for mathematical modeling of these mental processes). Each mental model-simulator projects a set of priming, top-down signals, representing the bottom-up signals expected from a particular object. The salient property of DL is that initial states of mental representations are vague and unconscious (or not fully conscious). In the processes of perception and cognition representations are matched to bottom-up signals and become more crisp and conscious. This is discussed in detail later along with references to experimental publications proving that this is a valid model for brain-mind processes of perception and cognition. Mathematical models of mental models-simulators characterize these mental models by parameters. Parameters describe object position, angles, lightings, *etc.* (In case of learning situations considered later, parameters characterize objects and relations making up a situation.) To summarize this highly simplified description of a visual system, the learning-perception process “matches” top-down and bottom-up activations by selecting “best” mental models-simulators and their parameters and fitting them to the corresponding sets of bottom-up signals. This DL process mathematically models multiple simulators running in parallel, each producing a set of priming signals for various expected objects.

Mathematical criteria of the “best” fit between bottom-up and top-down signals were given in [16,25,30,31]. They are similar to probabilistic or informatics measures. In the first case they represent probabilities that the given (observed) data or bottom-up signals correspond to representations-models (top-down signals) of particular objects. In the second case they represent information contained in representations-models about the observed data (in other words, information in top-down signals about bottom-up signals). These similarities are maximized over the model parameters. Results can be interpreted correspondingly as a maximum likelihood that models-representations fit sensory signals, or as maximum information in models-representations about the bottom-up signals. Both similarity measures account for all expected models and for all combinations of signals and models. Correspondingly, a similarity contains a large number of items, a total of *M^N^*, where *M* is a number of models and *N* is a number of signals; this huge number is the cause for the combinatorial complexity discussed previously.

Maximization of a similarity measure is a mathematical model of an unconditional drive to improve the correspondence between bottom-up and top-down signals (representations-models). In biology and psychology it was discussed as curiosity, a need to reduce cognitive dissonance, or a need for knowledge since the 1950s [42,43,44]. This process involves knowledge-related emotions evaluating satisfaction of this drive for knowledge [25,30,31,32,45,46]. In computational intelligence it is even more ubiquitous, every mathematical learning procedure, algorithm, or neural network maximizes some similarity measure. 

The DL learning process can be understood as both an artificial intelligence system or a cognitive model. Let us repeat, DL consists in estimating parameters of concept-models (mental representations) and associating subsets of bottom-up signals with top-down signals originating from these models-concepts by maximizing a similarity. Although a similarity contains combinatorially many items, DL maximizes it without combinatorial complexity [25,27,30,31,32,34,47] as follows. First, vague-fuzzy association variables are defined, which give a measure of correspondence between each signal and each model. They are defined similarly to the a posteriori Bayes probabilities, they range between 0 and 1, and as a result of learning they converge to the probabilities, under certain conditions. Often the association variables are close to bell-shapes.

The DL process is defined by a set of differential equations given in the above references; together with models discussed later it gives a mathematical description of perception and cognition processes, including the PSS simulators. To keep the review self-consistent we summarize these equations in Appendix. Those interested in mathematical details can read the Appendix. However, basic principles of DL can be adequately understood from a conceptual description and examples in this and following sections. As a mathematical model of perception-cognitive processes, DL is a process described by differential equations given in the Appendix; in particular, fuzzy association variables *f* associate bottom-up signals and top-down models-representations. Among unique DL properties is an autonomous dependence of association variables on models-representations: in the processes of perception and cognition, as models improve and become similar to patterns in the bottom-up signals, the association variables become more selective, more similar to delta-functions. Whereas initial association variables are vague and associate near all bottom-up signals with virtually any top-down model-representations, in the processes of perception and cognition association variables are becoming specific, “crisp”, and associate only appropriate signals. This is a process “from vague to crisp”, and also “from unconscious to conscious mental states” (The exact mathematical definition of crisp corresponds to values of *f* = 0 or 1; values of *f* in between 0 and 1 correspond to various degrees of vagueness.) The fact that “vague to crisp” is equivalent to “unconsciousness to conscious” has been experimentally demonstrated in [4].

DL processes mathematically model PSS simulators and not static amodal signals. Another unique aspect of DL is that it explains how logic appears in the human mind; how illogical dynamic PSS simulators give rise of classical logic, and what is the role of amodal symbols. This is discussed throughout the paper, and also in specific details in section 6.

An essential aspect of DL, mentioned above, is that associations between models and data (top-down and bottom-up signals) are uncertain and dynamic; their uncertainty matches uncertainty of parameters of the models and both change in time during perception and cognition processes. As the model parameters improve, the associations become crisp. In this way the DL model of simulator-processes avoids combinatorial complexity because there is no need to consider separately various combinations of bottom-up and top-down signals. Instead, all combinations are accounted for in the DL simulator-processes. Let us repeat that, initially, the models do not match the data. The association variables are not the narrow logical variables 0, or 1, or nearly logical, instead they are wide functions (across top-down and bottom-up signals). In other words, they are vague, initially they take near homogeneous values across the data (across bottom-up and top-down signals); they associate all the representation-models (through simulator processes) with all the input signals [25,30,33]. Here we conceptually describe the DL process as applicable to visual perception, taking approximately 160 ms, according to the reference below. Gradually, the DL simulator-processes improve matching, models better fit data, the errors become smaller, the bell-shapes concentrate around relevant patterns in the data (objects), and the association variables tend to 1 for correctly matched signal patterns and models, and 0 for others. These 0 or 1 associations are logical decisions. In this way, classical logic appears from vague states and illogical processes. Thus certain representations get associated with certain subsets of signals (objects are recognized and concepts formed logically or approximately logically). This process “from vague-to-crisp” that matches bottom-up and top-down signals has been independently conceived and demonstrated in brain imaging research to take place in human visual system [4,48]. Thus DL PSS simulators describe how logic appears from illogical processes, and actually model perception mechanisms of the brain-mind as processes from unconscious to conscious brain states. By connecting conscious and unconscious states DL resolves a long-standing difficulty of free will and explains that past difficulties related to the idea of free will are difficulties of logic, the mind and DL overcomes these difficulties [49,50].

Mathematical convergence of the DL process was proven in [25]. It follows that the simulator-process of perception or cognition assembles objects or concepts among bottom-up signals, which are most similar in terms of the similarity measure. Despite a combinatorially large number of items in the similarity, a computational complexity of DL is relatively low, it is linear in the number of signals, and therefore could indeed model physical systems, like a computer or brain. 

### 1.4. Example of DL, Object Perception in Noise

**Figure 1:**
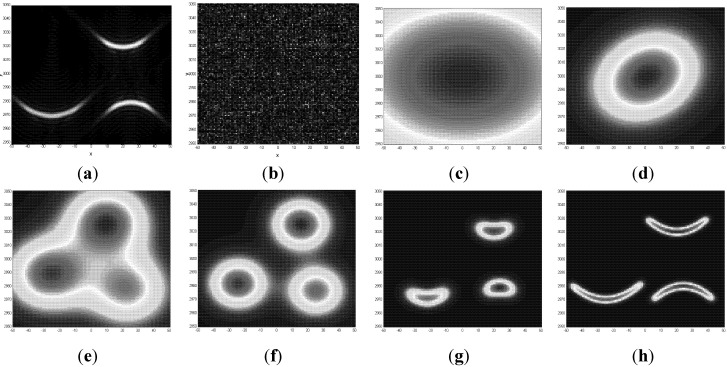
Finding “smile” and “frown” patterns in noise, an example of dynamic logic operation: (**a**) true “smile” and “frown” patterns are shown without noise; (**b**) actual image available for recognition (signals are below noise, signal-to-noise ratio is between ½ and ¼, 100 times lower than usually considered necessary); (**c**) an initial fuzzy blob-model, the vagueness corresponds to uncertainty of knowledge; (**d**) through (**h**) show improved models at various steps of dynamic logic (DL) (equation A3 are solved in 22 steps). Between stages (**d**) and (**e**) the algorithm tried to fit the data with more than one model and decided that it needs three blob-models to “understand” the content of the data. There are several types of models: One uniform model describing noise (it is not shown) and a variable number of blob-models and parabolic models, which number, location, and curvature are estimated from the data. Until about stage (**g**) the algorithm “thought” in terms of simple blob models, at (**g**) and beyond, the algorithm decided that it needs more complex parabolic models to describe the data. Iterations stopped at (**h**), when similarity (equation A1) stopped increasing.

The purpose of this section is to illustrate the DL perception processes, multiple simulators running in parallel as described above. We use a simple example, still unsolvable by other methods, (mathematical details are omitted, they could be found in [51]). In this example, DL searches for patterns in noise. Finding patterns below noise can be an exceedingly complex problem. If an exact pattern shape is not known and depends on unknown parameters, these parameters should be found by fitting the pattern model to the data. However, when the locations and orientations of patterns are not known, it is not clear which subset of the data points should be selected for fitting. A standard approach for solving this kind of problem, which has already been mentioned, is multiple hypotheses testing [20]; this algorithm exhaustively searches all logical combinations of subsets and models and is not practically useful because of CC. Nevertheless, DL successfully find the patterns under noise. In the current example, we are looking for “smile” and “frown” patterns in noise shown in Figure 1a without noise, and in Figure 1b with noise, as actually measured. Object signals are about 2–3 times below noise and cannot be seen by human visual system (it is usually considered that human visual system is better than any algorithm for perception of objects, therefore we emphasize that DL exceeds performance of human perception in this case because DL models work well with random noise, while human perception was not optimized by evolution for this kind of signals).

To apply DL to this problem, we used DL equations given in the Appendix. Specifics of this example are contained in models. Several types of models are used: parabolic models describing “smiles” and “frown” patterns (unknown size, position, curvature, signal strength, and number of models), circular-blob models describing approximate patterns (unknown size, position, signal strength, and number of models), and noise model (unknown strength). Exact mathematical description of these models is given in several references cited above.

The image size in this example is 100 × 100 points (*N* = 10,000 bottom-up signals, corresponding to the number of receptors in an eye retina), and the true number of models is 4 (3 + noise), which is not known. Therefore, at least *M* = 5 models should be fit to the data, to decide that 4 fits best. This yields complexity of logical combinatorial search, *M^N^* = 10^5000^; this combinatorially large number is much larger than the size of the Universe and the problem was considered unsolvable. Figure 1 illustrates DL operations: (a) true “smile” and “frown” patterns without noise; (b) actual image available for recognition; (c) through (h) illustrates the DL process, they show improved models at various steps of solving DL equation A3, total of 22 steps (noise model is not shown; figures (c) through (h) show association variables, f, for blob and parabolic models). By comparing (h) to (a) one can see that the final states of the models match patterns in the signal. Of course, DL does not guarantee finding any pattern in noise of any strength. For example, if the amount and strength of noise would increase 10-fold, most likely the patterns would not be found (this would provide an example of “falsifiability” of DL; however more accurate mathematical description of potential failures of DL algorithms is considered later). DL reduced the required number of computations from combinatorial 10^5000^ to about 10^9^. By solving the CC problem DL was able to find patterns under the strong noise. In terms of signal-to-noise ratio this example gives 10,000% improvement over the previous state-of-the-art. (We repeat that in this example DL actually works better than human visual system; the reason is that human brain is not optimized for recognizing these types of patterns in noise.) 

The main point of this example is that DL perception process, or PSS “simulator” is a process “from vague-to-crisp”, similar to visual system processes demonstrated in [4] (in that publication authors use the term “low spatial frequency” for what we call “vague” in Figure 1). 

We would also like to take this moment to continue the arguments from section 1.1, section 1.2, and to emphasize that DL is a fundamental and revolutionary improvement in mathematics [33,34]; it was recognized as such in mathematical and engineering communities; it is the theory that has suggested vague initial states; it has been developed for over 20 years; yet it might not be well known in cognitive science community. Those interested in a large number of mathematical and engineering applications of DL could consult given references and references therein. Here we would like to address two specific related concerns, first, if the DL algorithms are falsifiable, second, a possibility that Figure 1 example could be “lucky” or “erroneous”. We appreciate that some readers could be skeptical about 10,000% improvement over the state of the art. In mathematics there is a standard procedure for establishing average performance of detection (perception) and similar algorithms. It is called “operating curves” and it takes not one example, but tens of thousands examples, randomly varying in parameters, initial conditions, *etc.* The results are expressed in terms of probabilities of correct and incorrect algorithm performance (this is an exact mathematical formulation of the idea of “falsifiability” of an algorithm). These careful procedures demonstrated that Figure 1 represents an average performance of the DL algorithm [25,52,53].

### 1.5. The Knowledge Instinct (KI)

The word “instinct” fell out of favor in psychology and cognitive science, because of historical uncertainties of what it means. It was mixed up with “instinctual behavior” and other not well defined mechanisms and abilities. However, using a word “drive” is not adequate either, because fundamental inborn drives and culturally evolved drives are mixed up. In this section we follow instinctual-emotional theory of Grossberg and Levine [45] that gives succinct definition of instincts, enables mathematical modeling of these mechanisms underlying this review, corresponds to psychological, cognitive, and physiological data, and thus restores the scientific credibility of the word “instinct”. According to [45] instinct is an inborn mechanism that measures vital organism data and determines when these data are within or outside of safe regions. These results are communicated to decision-making brain regions (conscious or unconscious) by emotions (emotional neural signals), resulting in allocating resources to satisfying instinctual needs. Emotional neural signals also result in various physiological and psychological effects, however we would like to emphasize that for mathematical modeling and scientific understanding of the nature of instincts and emotions Grossberg-Levine theory is fundamental, whereas Damasio’s emotions as “bodily markers” are secondary. For some purposes it might be necessary to analyze physiological mechanisms of instincts, as well as physiological and psychological manifestations of emotions. However, within this review the Grossberg-Levine theory is a fundamental level of analysis.

A simplified example of the instinctual-emotional theory is an instinctual need for food. Special sensory-like physiological mechanisms measure sugar level in blood. When it drops below certain level an organism feels an emotion of hunger. Emotional neural signals indicate to decision-making parts of the brain that more resources have to be allocated to finding food. We have dozens of similar instinctual-emotional mechanisms: sensory-like measurements mechanisms and corresponding emotional signals.

Matching bottom-up and top-down signals, as mentioned, is the essence of perception and cognition processes, and constitutes an essential need for understanding the surrounding world. Models stored in memory as representations of past experiences never exactly match current objects and situations. Therefore thinking and even simple perception always require modifying existing models; otherwise the brain-mind would not be able to perceive the surroundings and the organism would not be able to survive. To survive, humans and higher animals have an inborn drive to fit top-down and bottom-up signals. Because the very survival of a higher animal or human depends on this drive, it is even more fundamental than drives for food or procreation; understanding the world around is a condition for satisfying all other instinctual needs. Therefore this drive for knowledge is called the knowledge instinct, KI [13,16,25,27,30]. 

This mechanism is similar to other instincts [13,25,30,45] in that our mind has a sensor-like mechanism that measures a similarity between top-down and bottom-up signals, between concept-models and sensory percepts. Brain areas participating in the knowledge instinct were discussed in [54]. As discussed in that publication, biologists considered similar mechanisms since the 1950s; without a mathematical formulation, however, its fundamental role in cognition was difficult to discern. All learning algorithms have some models of this instinct, maximizing correspondence between sensory input and an algorithmic internal structure (knowledge in a wide sense). According to the Grossberg-Levine instinct-emotion theory, satisfaction or dissatisfaction of every instinct is communicated to other brain areas by emotional neural signals. Emotional signals related to KI are felt as harmony or disharmony between our knowledge-models and the world. At lower levels of everyday object recognition these emotions are usually below the threshold of consciousness; at higher levels of abstract and general concepts this feeling of harmony or disharmony could be strong, as discussed in [13,28], it is a foundation of human higher mental abilities. Since Kant [55], emotions related to knowledge are called aesthetic emotions. Here we emphasize that they are related to every process of perception and cognition. They are “higher” emotions in the sense that they are related to knowledge, rather than to bodily needs; we would emphasize that this distinction is not fundamental in terms of mechanisms, all instinctual and emotional mechanisms involve brain. Yet for understanding human psychology it is a fundamental distinction. Their relations to higher cognitive abilities, to emotions of the beautiful and sublime are discussed later.

Mathematical modeling of perception and thinking emphasized fundamental nature of KI: all mathematical algorithms for learning have some variation of this process, matching bottom-up and top-down signals. Without matching previous models to the current reality we will not perceive objects, or abstract ideas, or make plans. This process involves learning-related emotions evaluating satisfaction of KI [25,27,30,46,56].

### 1.6. Emotions of Beautiful and Sublime

DL model of KI inherently involves emotional signals related to satisfaction or dissatisfaction of KI. These emotions are modeled by changes in the similarity between bottom-up and top-down signals, in other world by KI satisfaction. We perceive these emotions as feelings of harmony or disharmony between our knowledge and the world or within the knowledge; these emotions related to knowledge are called aesthetic emotions [55]. KI and aesthetic emotions drive the brain-mind to improve mental models-concepts for better correspondence to surrounding objects and events. This section relates aesthetic emotions to the beautiful and sublime according to [25,28,30,34,57,58,59,60,61,62,63,64].

Cognitive science and psychology for decades have been at a complete loss when trying to identify cognitive functions of the highest human abilities, the most important and cherished ability to create and perceive the beautiful. Its role in the working of the mind was not understood. Aesthetic emotions discussed above are often below the level of consciousness at lower levels of the mind hierarchy. Simple harmony is an elementary aesthetic emotion related to improvement of mental models of objects. Higher aesthetic emotions are related to the development and improvement of more complex “higher” models at higher levels of the mind hierarchy. At higher levels, when understanding important concepts, aesthetic emotions reach consciousness.

Models at higher levels of the mind hierarchy are more general than lower-level models; they unify knowledge accumulated at lower levels. This is the *purpose* for which neural mechanisms of concepts emerged in genetic and cultural evolution, and this purpose in inseparable from the content of these models. The highest forms of aesthetic emotions are related to the most general and most important models near the top of the mind hierarchy. The purpose of these models is to unify our entire life experience. This conclusion is identical to the main idea of Kantian aesthetics. According to Kantian analysis among the highest models are models of the meaning of our existence, of our purposiveness or intentionality. KI drives us to develop these models. The reason is in the two sides of knowledge: on one hand knowledge consists in detailed models of objects and events generating bottom-up signals at every hierarchical level, on the other, knowledge is a more general and unified understanding of lower-level models at higher levels, generating top-down signals. These two sides of knowledge are related to viewing the knowledge hierarchy from bottom up or from top down. In the top-down direction, models strive to differentiate into more and more detailed models accounting for every detail of the reality. In the bottom-up direction, models strive to make a larger sense of the detailed knowledge at lower levels. In the process of cultural evolution, higher, general models have been evolving with this purpose, to make more sense, to create more general meanings. In the following sections we consider mathematical models of this process of cultural evolution, in which top mental models evolve. The most general models, at the top of the hierarchy, unify all our knowledge and experience. The mind perceives them as the models of meaning and purpose of existence. In this way KI theory corresponds to Kantian analysis. 

Everyday life gives us little evidence to develop models of meaning and purposiveness of our existence. People are dying every day and often from random causes. Nevertheless, belief in one’s purpose is essential for concentrating will and for survival. Is it possible to understand psychological contents and mathematical structures of models of meanings and purpose of human life? It is a challenging problem yet DL gives a foundation for approaching it.

Consider a simple experiment: remember an object in front of your eyes. Then close eyes and recollect the object. The imagined object is vague, not as crisp as this same object a moment ago, when perceived with opened eyes. Imaginations of objects are top-down projections of object representations on the visual cortex. We conclude that mental representations-models of everyday objects are vague (as modeled by DL). We can conclude that models of abstract situations, higher in the hierarchy, which cannot be perceived with “opened eyes”, are much vaguer. Even much vaguer have to be models of the purpose of life at the top of the hierarchy. As mentioned, everyday life gives us no evidence that such a meaning and purpose exist at all. And many people do not believe that life has a meaning. When we ask our colleagues-scientists if life has a meaning, most protest against such a nebulous, indefinable, and seemingly unscientific idea. However, nobody would agree that his or her personal life is as meaningless as a piece of rock at a road wayside.

Is there a scientific way to resolve this contradiction? This is exactly what we intend to do in this section with the help of DL mathematical models and recent results of neuro-psychological experiments. Let us go back again to the closed eye experiment. Vague imaginations with closed eyes cannot be easily recollected when eyes are opened. Vague states of mental models are not easily accessible to consciousness. To imagine vague objects we should close eyes. Can we “close mental eyes” that enable cognition of abstract models? Later we consider mathematical models of this process. Here we formulate the conclusions. “Mental eyes” enabling cognition of abstract models involve language models of abstract ideas. These language models are results of millennia of cultural evolution. High-level abstract models are formulated crisply and consciously in language. To significant extent they are cultural constructs, and they are different in different cultures. Every individual creates cognitive models from his or her experience guided by cultural models stored in language. Whereas language models are crisp and conscious, cognitive models are vague and less conscious. Few individuals in rare moments of their lives can understand some aspects of reality beyond what has been understood in culture over millennia and formulated in language. In these moments “language eyes” are closed and an individual can see “imagined” cognitive images of reality not blinded by culturally received models. Rarely these cognitions better represent reality than millennial cultural models. And even rarer these cognitions are formulated in language so powerfully that they are accepted by other people and become part of language and culture. This is the process of cultural evolution. We will discuss it in more details later. 

Understanding the meaning and purpose of one’s life has been important for survival millions of years ago and is important for achieving higher goals in contemporary life. Therefore all cultures and all languages forever have been formulating contents of these models. And the entire humankind has been evolving toward better understanding of the meaning and purpose of life. Those individuals and cultures that do not succeed are handicapped in survival and expansion. But let us set aside cultural evolution for later sections and return to how an individual perceives and feels his or her models of the highest meaning.

As discussed, cognitive models at the very top of the mind hierarchy are vague and unconscious. Even so many people are versatile in talking about these models, and many books have been written about them, cognitive models that correspond to the reality of life are vague and unconscious. Some people, at some points in their life, may believe that their life purpose is finite and concrete, for example to make a lot of money, or build a loving family and bring up good children. These crisp models of purpose are cultural models, formulated in language. Usually they are aimed at satisfying powerful instincts, but not KI and they do not reflect the highest human aspirations. Reasons for this perceived contradiction are related to interaction between cognition and language that we have mentioned and will be discussing in more details later. Anyone who has achieved a finite goal of making money or raising good children knows that this is not the end of his or her aspirations. The psychological reason is that everyone has an ineffable feeling of partaking in the infinite, while at the same time knowing that one’s material existence is finite. This contradiction cannot be resolved. For this reason cognitive models of our purpose and meaning cannot be made crisp and conscious, they will forever remain vague, fuzzy, and mostly unconscious. 

As discussed, better understanding of what the model is about leads to satisfaction of KI, and to corresponding aesthetic emotions. Higher in the hierarchy the models are vague, less conscious and emotional contents of mental states are less separated from their conceptual contents. At the top of the mind hierarchy, the conceptual and emotional contents of cognitive models of the meaning of life are not separated. In those rare moments when one improves these models, improves understanding of the meaning of one’s life, or even feels assured that the life has meaning, he or she feels emotions of the beautiful, the aesthetic emotion related to satisfaction of KI at the highest levels. 

These issues are not new; philosophers and theologians expounded them from time immemorial. The DL-KI theory gives us a scientific approach to the eternal quest for the meaning. We perceive an object or a situation as beautiful, when it stimulates improvement of the highest models of meaning. Beautiful is what “reminds” us of our purposefulness. This is true about perception of beauty in a flower or in an art object. Just an example, R. Buckminster Fuller, an architect, best known for inventing the geodesic dome wrote: “When I’m working on a problem, I never think about beauty. I think only how to solve the problem. But when I have finished, if the solution is not beautiful, I know it is wrong.” Similar things were told about scientific theories by Einstein and Poincare, emphasizing that the first proof of a scientific theory is its beauty. The KI theory explanation of the nature of the beautiful helps understanding an exact meaning of these statements and resolves a number of mysteries and contradictions in contemporary aesthetics.

Emotions of spiritually sublime are similar to and different from emotions of the beautiful. Emotions of the beautiful are related to *understanding* contents of the highest concepts of meaning. Emotions of spiritually sublime are related to *behavior* that could make the meaning and beautiful a part of one’s life [59]. This is the foundation of all religions. It is unfortunate that this foundation has been almost forgotten and often hidden behind fog of pragmatic usefulness of church life, differences among churches, historical enmity between religion and science, neglect and often contempt by scientists toward religion. This explanation is a bridge required by culture to connect science and religion.

Finishing scientific discussion of the beautiful and sublime, we would like to emphasize again that these are emotions related to knowledge at the top of the mind hierarchy, the knowledge of the life meaning. It is governed by KI, not by sex and instinct for procreation. Sexual instinct is among the strongest of our bodily instincts, and it makes use of all our abilities, including knowledge, beauty, and strivings for sublime. And yet the ability for feeling and creating the beautiful and sublime are related not to sexual instinct but to the instinct for knowledge.

A fundamental conclusion from this section is that the brain-mind is not logical, whereas intuition of most lay people and scientists that brain-mind is mostly logical is wrong. This conclusion is difficult to accept and to make sense of for non-mathematicians as well as for many mathematicians. Future developments in psychology and cognitive science require no less than a revolution in scientific intuition and thinking. And the current review might help in this process.

## 2. DL of PSS: Perceptual Cognition and Simulators

### 2.1. Introduction. PSS, Challenge of Computational Model

Let us repeat that PSS is a well accepted and intuitively clear cognitive (non-mathematical) model of perception and cognition. Therefore connecting the previous discussion with PSS might help in making the revolutionary step toward new intuition of the brain-mind. PSS grounds cognition in perception [21]. “Grounded cognition … rejects the standard view that amodal symbols represent knowledge in semantic memory” [21]. PSS emphasized the roles of simulation in cognition. “Simulation is the reenactment of perceptual, motor, and introspective states acquired during experience with the world, body, and mind … when knowledge is needed to represent a category (e.g., chair), multimodal representations captured during experiences … are reactivated to simulate how the brain represented perception, action, and introspection associated with it.” Simulation is an essential computational mechanism in the brain. The best known case of these simulation mechanisms is mental imagery [2,3]. According to PSS cognition supports action. Simulation is a central mechanism of PSS, yet rarely, if ever, they recreate full experiences. Using the mechanism of simulators, which approximately correspond to concepts and types in amodal theories, PSS implements the standard symbolic functions of type-token binding, inference, productivity, recursion, and propositions. Using these mechanisms PSS retains the symbolic functionality. “Thus, PSS is a synthetic approach that integrates traditional theories with grounded theories” [21,65,66].

According to Barsalou, during the Cognitive Revolution in the middle of the last century, cognitive scientists were inspired by new forms of representation “based on developments in logic, linguistics, statistics, and computer science”. They adopted amodal representations, such as feature lists, semantic networks, and frames [67]. Little empirical evidence supports amodal symbolic mechanisms [21]. It seems that amodal symbols were adopted largely because they promised to provide “elegant and powerful formalisms for representing knowledge, because they captured important intuitions about the symbolic character of cognition, and because they could be implemented in artificial intelligence”. As we have discussed these promises were unfulfilled due to fundamental mathematical difficulties.

There is a number of past and ongoing developments of computational implementations of PSS [68,69] and references therein. Yet, computational models for PSS [21,61] require new mathematical methods of DL different from traditional artificial intelligence, pattern recognition, or connectionist methods. We discussed the reason: the traditional methods encountered combinatorial complexity (CC), an irresolvable computational difficulty, when attempting to model complex systems. Cognitive modeling requires learning combinations of perceptual features and objects or events [17,18,23,24,28,31,70,71,72].

In this review we discuss a realistic and scalable mathematical model of perception, cognition, grounded symbols and formalization of PSS based on a new computational technique of DL as developed in [23,24]. Although the developed mathematical formalism is quite general, here we first concentrate on just one example of PSS mechanism: a mathematical description of models and simulators for forming and enacting representations of situations (higher level symbols) from perceptions of objects (lower level symbols), and then we discuss its general applicability. In addition to simulators, we consider concepts, grounding, binding, dynamic aspect of PSS (DIPSS), abstract concepts, the mechanism of amodal symbols within PSS, and the role of logic. The mathematical models of PSS serving as a foundation for this discussion enabled establishing limits of PSS as conceived by Barsalou [21], and later we discuss necessary modifications and extension of PSS.

### 2.2. Initial Relation of DL and PSS

Section 1.4 illustrated DL for recognition of simple objects in noise, a case complex and unsolvable for prior state-of-the-art algorithms, still too simple to be directly relevant for PSS. Here we consider a problem of situation learning, assuming that object recognition has been solved. In computational image recognition this is called “situational awareness” and it is a long-standing unsolved problem. The principled difficulty is that every situation includes many objects that are not essential for recognition of this specific situation; in fact there are many more “irrelevant” or “clutter” objects than relevant ones. Let us dwell on this for a bit. Objects are spatially-limited material things perceptible by senses. A situation is a collection of contextually related objects that tend to appear together and are perceived as meaningful, e.g., an office, a dining room. The requirement for contextual relations and meanings makes the problem mathematically difficult. Learning contexts comes along with learning situations; it reminds of the problem of a chicken and egg. We subliminally perceive many objects, most of which are irrelevant, e.g., a tiny scratch on a wall, which we learn to ignore. Combinations of even a limited number of objects exceed what is possible to learn in a single lifetime as meaningful situations and contexts (e.g., books on a shelf) from random sets of irrelevant objects (e.g., a scratch on a wall, a book, and a pattern of tree branches in a window). Presence of hundreds (or even dozens) irrelevant objects makes learning by a child of mundane situations a mathematical mystery. In addition, we constantly perceive large numbers of different objects and their combinations, which do not correspond to anything worth learning and we successfully learn to ignore them. 

An essential part of learning-cognition is to learn which sets of objects are important for which situations (contexts). The key mathematical property of DL that made this solution possible, same as in the previous section, is a process “from vague-to-crisp”. Concrete crisp models-representations of situations are formed from vague models in the process of learning (or cognition-perception). We illustrate below how complex symbols, situations, are formed by situation-simulators from simpler perceptions, objects, which are simpler perceptual symbols, being formed by simulators at “lower” levels of the mind, comparative to “higher” situation-simulators. Situation-simulators operate on mental representations of situations (such as described by PSS), which are dynamic and vague assemblages of situations from imagery (and other modalities), bits and pieces along with some relations among them perceived at lower levels. These pieces and relations may come from different past perceptions, not necessarily from a single perceptual mode, and not necessarily stored in a contiguous parts of the brain. The dynamic process of DL-PSS-simulation, which assembles these bits into situations attempting to match those before the eyes, is mostly unconscious. We will discuss in details in section 6 that these are perceptual symbols as described in [21]. DL mathematically models PSS simulators, processes that match bottom-up perceptions with top-down signals, assemble symbols in cognition-perception, and assemble conceptual representations by recreating patterns of activation in sensorimotor brain areas (as discussed later in the paper). An essential mechanism of DL cognition-perception is a process of simulation of perceptual imagination-cognitions; these situation-symbols are simulated from simpler perceptions-objects (we repeat that these simulations-imaginations are not limited to imagery, and are mostly unconscious). And the same mechanism can simulate plans and more complex abstract thoughts, as discussed in later sections. Thus, in the following sections we demonstrate that DL successfully models PSS simulators, in this case simulators of situations and leads to learning of situations, while discarding irrelevant objects.

### 2.3. DL for Learning Situations

In a simplified problem considered here, the task is for an intelligent agent (a child) to learn to recognize certain situations in the environment; while it is assumed that a child has learned to recognize objects. In real life a child learns to recognize situations, to some extent, in parallel with recognizing objects. But for simplicity of the illustration examples and discussions below, we consider a simplified case of objects being already known. For example, situation “office” is characterized by the presence of a chair, a desk, a computer, a book, a book shelf. Situation “playground” is characterized by the presence of a slide, a sandbox, *etc.* The principal difficulty is that many irrelevant objects are present in every situation. (This child learning is no different mathematically from an adult recognition.)

In the example below, *D*_o_ is the total number of objects that the child can recognize in the world (it is a large number). In every situation he or she perceives *D*_p_ objects. This is a much smaller number compared to *D*_o_. Each situation is also characterized by the presence of *D*_s_ objects essential for this situation (*D*_s_ < *D*_p_). Normally nonessential objects are present and *D*_s_ is therefore less than *D*_p_. The sets of essential objects for different situations may overlap, with some objects being essential to more than one situation. The real life learning is sequential as a child is exposed to situations one at a time. DL can handle this, but in this paper we consider the data about all the situations available at the time of learning. A mathematical formulation is given in the Appendix. Here we discuss the problem conceptually and illustrate the solution in the following figures.

In the following example we set the total number of recognizable objects equal to 1000 (*D*_o_ = 1000). The total number of objects perceived in a situation is set to 50 (*D*_p_ = 50). The number of essential objects is set to 10 (*D*_s_ = 10). The number of situations to learn (*M* − 1) is set to 10. Note that the true identities of the objects are not important in this simulation so we simply use object indexes varying from 1 to 1000 (this index points to neural signals corresponding to a specific object-simulators). The situation names are also not important and we use situation indexes (this index points to neural signals corresponding to a specific situation-simulators). We would emphasize that the use of numbers for objects and situation, while may seem consistent with amodal symbols, in fact is nothing but notations. We repeat that the principled differences between The DL-PSS and amodal systems are mechanisms in the brain and their modeling, not mathematical notations. Among these mechanisms are simulators, mathematically described by DL. Let us repeat, amodal symbols are governed by classical logic, which is static and faces CC. DL is a process and overcomes CC. DL operates on PSS representations (models described in the Appendix), which are vague collections of objects (some of these objects could also be vague, not completely assembled yet representations). Another principled difference is interaction between perceptual-based bottom-up and top-down neural fields. In this review we consider object perception and situation perception in different sections, but of course the real mind-brain operates continuously, “objects” in this section are neural signals sent to situation-recognition brain area (and corresponding simulators) by excited neuron fields corresponding to models of recognized-objects (partially, as described in section 2; and as discussed, these signals are being sent before objects are fully recognized, while object simulators are still running).

**Figure 2:**
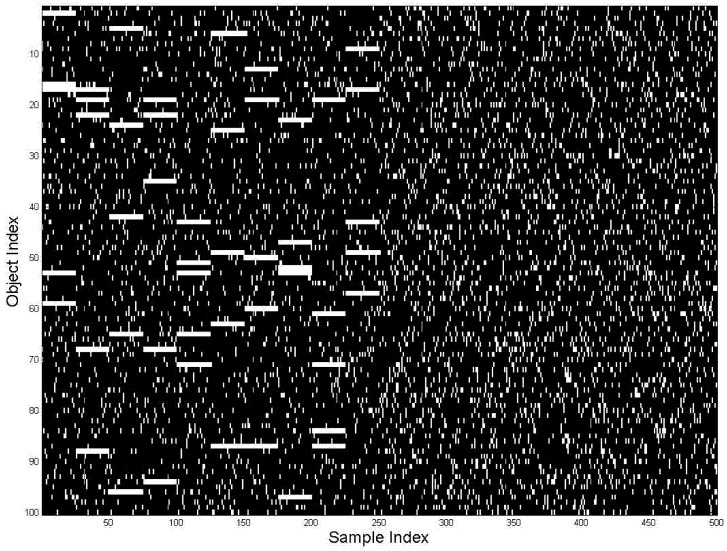
Generated data; object index is along vertical axes and situation index is horizontal. The perceptions (data samples) are sorted by situation index (horizontal axis); this makes visible the horizontal lines for repeated objects.

The data for this example are generated by first randomly selecting *D*_s_ = 10 specific objects for each of the 10 groups of objects, allowing some overlap between the groups (in terms of specific objects). This selection is accomplished by setting the corresponding probabilities *p*_mi_ = 1. Next we add 40 more randomly selected objects to each group (corresponding to *D*_p_ = 50). We also generate 10 more random groups of 50 objects to model situations without specific objects (noise); this is of course equivalent to 1 group of 500 random objects. We generate *N*′ = 800 perceptions for each situation resulting in *N* = 16,000 perceptions (data samples, *n* = 1 … 16,000) each represented by 1000-dimensional vector of observed data. These data are shown in Figure 2 sorted by situations.

**Figure 3:**
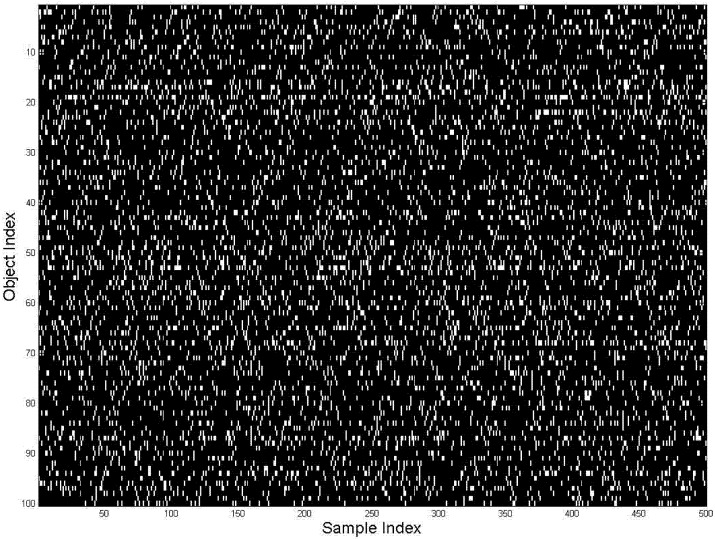
Data, same as Figure 2, randomly sorted by situations (horizontal axis), as available to the DL algorithm for learning.

Then the samples are randomly permuted, according to randomness of real life perceptual situations, in Figure 3. The horizontal lines disappear; the identification of repeated objects becomes nontrivial. An attempt to learn groups-situations (the horizontal lines) by inspecting various horizontal sortings (until horizontal lines would become detectable) would require *M^N^* = 10^16000^ inspections, which is of course impossible. This CC is the reason why the problem of learning situations has been standing unsolved for decades. By overcoming CC, DL can solve this problem as described in the Appendix and is illustrated below.

The DL algorithm is initiated similarly to section 2 by defining 20 situational models (an arbitrary selection, given actual 10 situations) and one random noise model to give a total of *M* = 21 models (in section 1.4, Figure 1 models were automatically added by DL as required; here we have not done this because it would be too cumbersome to present results). The models are initialized by assigning random probability values to the elements of the models. These are the initial vague perceptual models, which assign all objects to all situations. 

**Figure 4:**
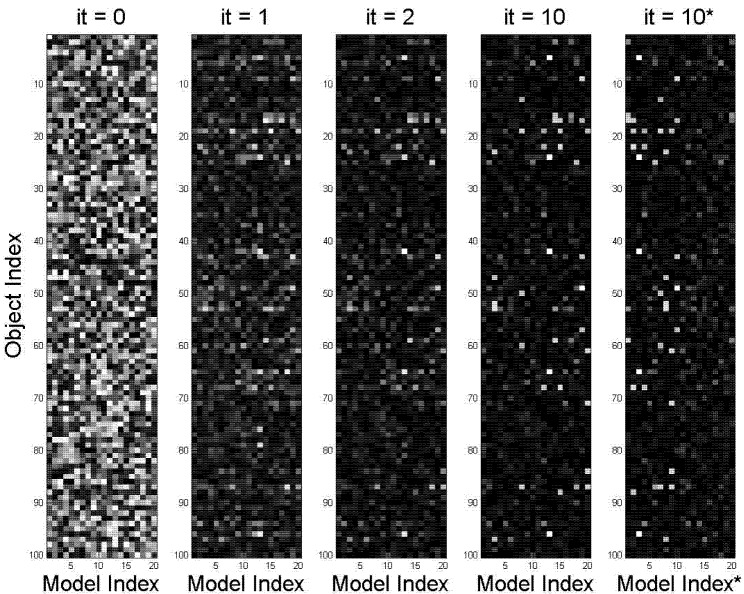
DL situation learning. Situation-model parameters converge close to true values in three steps.

Figure 4 illustrates the initialization and the iterations of the DL algorithm (the first 3 steps of solving DL equations). Each subfigure displays the probability model-vector for each of the 20 models. The vectors have 1000 elements corresponding to objects (vertical axes). The values of each vector element are shown in gray scale. The initial models assign nearly uniformly distributed probabilities to all objects. 

**Figure 5:**
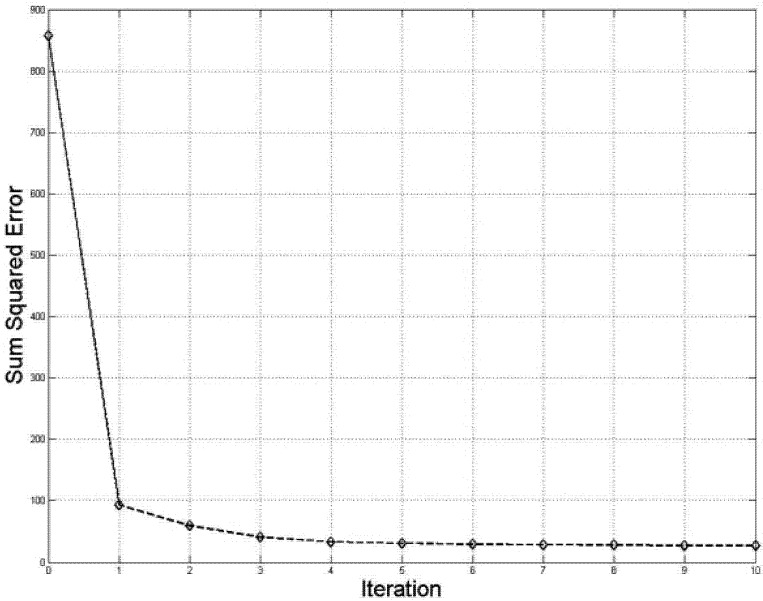
Errors of DL learning are quickly reduced in 3–4 steps, iterations continue until average error reached predetermined threshold of 0.05 (10 steps).

**Figure 6:**
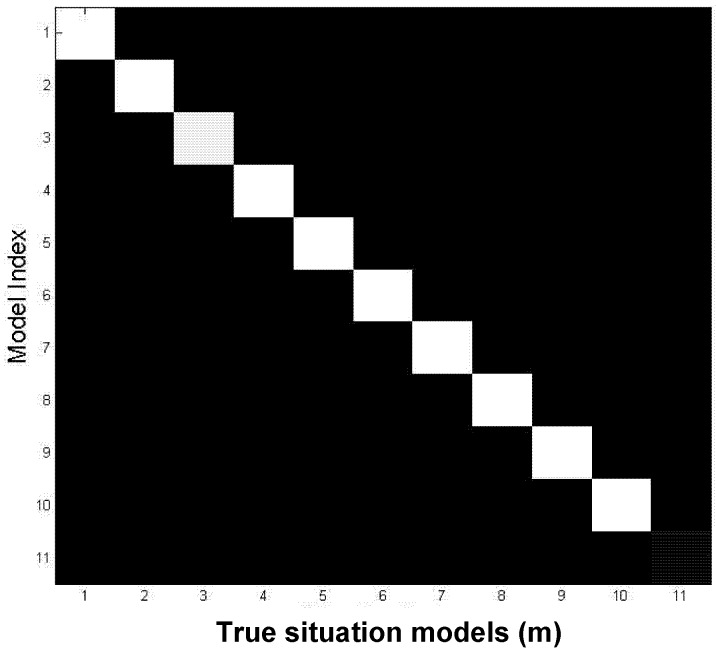
Correct associations are near 1 (diagonal, except noise) and incorrect associations are near 0 (off-diagonal).

The horizontal axes are the model index changing from 1 to 20. The noise model is not shown. As the algorithm progresses, situation grouping improves, and only the elements corresponding to repeating objects in “real” situations keep their high values, the other elements take low values. By the third iteration the 10 situations are identified by their corresponding models. The other 10 models converge to more or less random low-probability vectors. This fast and accurate convergence can be seen from Figure 5 and Figure 6. 

Again, as in section 2, learning of perceptual situation-symbols has been accomplished due to the DL process-simulator, which simulated internal model-representations of situations to match patterns in bottom-up.

The correct associations on the main diagonal in Figure 6 are 1 (except noise model, which is spread among 10 computed noise models, and therefore equals 0.1) and off-diagonal elements are near 0 (incorrect associations, corresponding to small errors shown in Figure 5). In [22,23,24] we discussed why errors in Figure 5 do not converge exactly to 0. The reason is numerical, and if desirable smaller values could have been obtained with few more iterations. Figure 6 demonstrates that nevertheless, convergence to the global maximum was achieved (the exactly known solution in terms of learning the correct situations). 

## 3. DL and PSS

### 3.1. Simulators, Concepts, Grounding, Binding, and DL

As described previously, PSS grounds perception, cognition, and high-level symbol operation in modal symbols, which are ultimately grounded in the corresponding brain systems. Previous section provides an initial development of formal mathematical description suitable for PSS: the DL process “from vague-to-crisp” models PSS simulators. We have considered just one subsystem of PSS, a mechanism of learning, formation, and recognition of situations from objects making up the situations. (More generally, the formalized mechanism of simulators includes recognition of situations by recreating patterns of activations in sensorimotor brain areas, from objects, relations, and actions making up the situations). The mind’s representations of situations are symbol-concepts of a higher level of abstractness than symbol-objects making them up. The proposed mathematical formalism can be advanced straightforwardly to “higher” levels of more and more abstract concepts. However, as we discuss in the following sections such application to abstract concepts requires an additional grounding in language [73,74] as we consider in the next sections. 

The proposed mathematical formalism can be similarly applied at a lower level of recognizing objects as constructed from their parts; mathematical techniques of section 1 and section 2 can be combined to implement this PSS object recognition idea as described in [21]. Specific models considered in section 1 are likely to be based on inborn mechanisms specific to certain aspects of various sensor and motor modalities; general models of section 2 can learn to represent and recognize objects as collections of multi-modal perceptual features and relations among them. In both cases principal mechanisms of object perception such as discussed in [75] can be modeled, either as properties of object models, or as relations between perceptual features. Since relations specific to object recognition, according to this reference are learned in infancy, the mechanism of section 2 seems appropriate (it models learning of relations, whereas models in section 1 do not readily contain mechanisms of learning of all their structural aspects and are more appropriate to modeling inborn mechanisms). Object representations, as described by Barsalou are not similar to photographs of specific objects, but similar to models in Figure 4 are more or less loose and distributed (among modalities) collections of features (determined in part by inborn properties of sensor and motor organs) and relations. 

We note that the described theory, by modeling the simulators, also mathematically models productivity of the mind concept-simulator system. The simulated situations and other concepts are used not only in the process of matching bottom-up and top-down signals for learning and recognizing representations, but also in the motor actions, and in the processes of imagination and planning.

Modeling situations in PSS as a step toward general solution of the binding problem is discussed in [76]. DL provides a general approach to the binding problem similar to the “corkboard” approach described in [77]. That publication also discusses the role of context similar to the DL scene modeling. Here we would emphasize two mechanisms of binding modeled in the developed theory. First, binding is accomplished hierarchically: e.g., object representations-simulators bind features into objects, similarly situation representations-simulators bind objects into situations, *etc.* Second, binding is accomplished by relations that are learned similarly to objects and “reside” at the same level in the hierarchy of the mind with the bound entities. These two types of binding mechanisms is another novel prediction of the DL theory that could be tested experimentally.

Below we discuss other relationships between the mathematical DL procedures of previous sections and the fundamental ideas of PSS. Section 1 concentrated on the principal mathematical difficulty experienced by all previous attempts to solve the problem of complex symbol formation from less complex symbols, the combinatorial complexity (CC). CC was resolved by using DL, a mathematical theory, in which learning begins with vague (non-specific) symbol-concepts, and in the process of learning symbol-concepts become concrete and specific. Learning could refer to a child’s learning, which might take days or months or an everyday perception and cognition, taking approximately 1/6th of a second (in the latter case, learning refers to the fact that every specific realization of a concept in the world is different in some respects from any previous occurrences, therefore learning-adaptation is always required; in terms of PSS, a simulator always have to re-assemble the concept). In the case of learning situations as compositions of objects, the initial vague state of each situation-symbol is a nearly random and vague collection of objects, while the final learned situation consists of a crisp collection of few objects specific to this situation. This specific of the DL process “from vague-to-crisp” is a prediction that can be experimentally tested, and we return to this later. In the learning process random irrelevant objects are “filtered out”, their probability of belonging to a concept-situation is reduced to zero, while probabilities of relevant objects, making up a specific situation is increased to a value characteristic of this object being actually present in this situation. 

Relation of this DL process to PSS is now considered. First we address concepts and their development in the brain. According to [61],

“The central innovation of PSS theory is its ability to implement concepts and their interpretative functions using image content as basic building blocks.”

This aspect of PSS theory is implemented in DL in a most straightforward way. Concept-situations in DL are collections of objects (symbol-models at lower levels, which are neurally connected to neural fields of object-images). As objects are perceptual entities-symbols in the brain, concept-situations are collections of perceptual symbols. In this way situations are perceptual symbols of a higher order complexity than object-symbols, they are grounded in perceptual object-symbols (images), and in addition, their learning is grounded in perception of images of situations. A PSS mathematical formalization of abstract concepts [78], not grounded in direct perceptions, is considered in the next section. Here we just mention that the proposed model is applicable to higher levels, “beyond” object-situations; it is applicable to modeling interactions between bottom-up and top-down signals at every level.

Barsalou [79] has described development of concepts in the brain as forming collections of correlated features. This is explicitly implemented in the DL process described in section 3. The developed mathematical representation corresponds to multimodal and distributed representation in the brain. It has been suggested that a mathematical set or collection is implemented in the brain by a population of conjunctive neurons [80].

DL learning and perception-cognition processes are mathematical models of PSS simulators. DL symbol-situations are not static collections of objects but dynamic processes. In the process of learning they “interpret individuals as tokens of the type” [72]. They model multi-modal distributed representations (including motor programs) as described in the reference. 

The same DL mathematical procedure can apply to perception of a real situation in the world as well as an imagined situation in the mind. This is the essence of imagination. Models of situations (probabilities of various objects belonging to a situation, and objects attributes, such as their locations) can depend on time, in this way they are parts of simulators accomplishing cognition of situations evolving in time. If “situations” and “time” pertain to the mind’s imaginations, the simulators implement imagination-thinking process, or planning. 

Usually we perceive-understand a surrounding situation, while at the same time thinking and planning future actions and imagine consequences. This corresponds to running multiple simulators in parallel. Some simulators support perception-cognition of the surrounding situations as well as ongoing actions, they are mathematically modeled by DL processes that converged to matching internal representations (types) to specific subsets in external sensor signals (tokens). Other simulators simulate imagined situations and actions related to perceptions, cognitions, and actions, produce plans, *etc.*

Developed here DL modeling of PSS models mathematically what Barsalou [71] called dynamic interpretation of PSS (DIPSS). DIPSS is fundamental to modeling abstraction processes in PSS. Three central properties of these abstractions are type-token interpretation; structured representation; and dynamic realization. Traditional theories of representation based on logic model interpretation and structure well but are not sufficiently dynamical. Conversely, connectionist theories are dynamic but are inadequate at modeling structure. PSS addresses all three properties. Similarly, the DL mathematical process developed here addresses all three properties. In type-token relations “propositions are abstractions for properties, objects, events, relations and so forth. After a concept has been abstracted from experience, its summary representation supports the later interpretation of experience.” Correspondingly in the developed mathematical approach, DL models a situation as a loose collection of objects and relations. Its summary representation (the initial model) is a vague and loose collection of property and relation simulators, which evolves-simulates representation of a concrete situation in the process of perception of this concrete situation according to DL. This DL process involves structure (initial vague models) and dynamics (the DL process). 

### 3.2. Perceptual *vs.* Amodal Symbols in DL and PSS

Since any mathematical notation may look like an amodal symbol, in this section we discuss the roles of amodal *vs.* perceptual symbols in DL and PSS. This would require clarification of the word symbol. We touch on related philosophical and semiotic discussions and relate them to mathematics of DL and to PSS. For the sake of brevity within this review we limit discussions to the general interest, emphasizing connections between DL, perceptual, and amodal symbols; extended discussions of symbols can be found in [31,81,82,83]. Kovalerchuk *et al.* discussed relationships of DL to other types of logic [84]. We also summarize here related discussions scattered throughout the review.

“Symbol is the most misused word in our culture” [85]. Why the word “symbol” is used in such a different way: to denote trivial objects, like traffic signs or mathematical notations, and also to denote objects affecting entire cultures over millennia, like Magen David, Swastika, Cross, or Crescent? Let us compare in this regard opinions of two founders of contemporary semiotics, Charles Peirce [86] and Ferdinand De Saussure [87]. Peirce classified signs into symbols, indexes, and icons. Icons have meanings due to resemblance to the signified (objects, situations, *etc.*), indexes have meanings by direct connection to the signified, and symbols have meaning due to arbitrary conventional agreements. Saussure used different terminology, he emphasized that signs receive meanings due to arbitrary conventions, whereas symbol implies motivation. It was important for him that motivation contradicted arbitrariness. Peirce concentrated on the process of sign interpretation, which he conceived as a triadic relationship of sign, object, and interpretant. Interpretant is similar to what we call today a representation of the object in the mind. However, this emphasis on interpretation was lost in the following generation of scientists. This process of “interpretation” is close to the DL processes and PSS simulators. We therefore follow Saussurean designation of symbol as a motivated process. Motivationally loaded interpretation of symbols was also proposed by Jung [88]. He considered symbols as processes bringing unconscious contents to consciousness. Similar are roles of PSS simulators and DL processes. (Motivated in DL means in particular related to drives, emotions).

In the development of scientific understanding of symbols and semiotics, the two functions, understanding the language and understanding the world, have often been perceived as identical. This tendency was strengthened by considering logical rules to be the mechanism of both, language and cognition. According to Russell [89], language is equivalent to axiomatic logic, “[a word-name] merely to indicate what we are speaking about; [it] is no part of the fact asserted … it is merely part of the symbolism by which we express our thought.” Hilbert [37] was sure that his logical theory also describes mechanisms of the mind, “The fundamental idea of my proof theory is none other than to describe the activity of our understanding, to make a protocol of the rules according to which our thinking actually proceeds.” Similarly, logical positivism centered on “the elimination of metaphysics through the logical analysis of language”—according to Carnap [90] logic was sufficient for the analysis of language. As discussed in section 2.2, this belief in logic is related to functioning of human mind, which is conscious about the final states of DL processes and PSS simulators; these final states are perceived by our minds as approximately logical amodal symbols. Therefore we identify amodal symbols with these final static logical states, signs.

DL and PSS explain how the mind constructs symbols, which have psychological values and are not reducible to arbitrary logical amodal signs, yet are intimately related to them. In section 3 we have considered objects as learned and fixed. This way of modeling objects indeed is amenable to interpreting them as amodal symbols-signs. Yet, we have to remember that these are but final states of previous simulator processes, perceptual symbols. Every perceptual symbol-simulator has a finite dynamic life, and then it becomes a static symbol-sign. It could be stored in memory, or participate in initiating new dynamical perceptual symbols-simulators. This infinite ongoing dynamics of the mind-brain ties together static signs and dynamic symbols. It grounds symbol processes in perceptual signals that originate them; in turn, when symbol-processes reach their finite static states-signs, these become perceptually grounded in symbols that created them. We become consciously aware of static sign-states, express them in language and operate with them logically. Then, outside of the mind-brain dynamics, they could be transformed into amodal logical signs, like marks on a paper. Dynamic processes—symbols-simulators are usually not available to consciousness. These PSS processes involving static and dynamic states are mathematically modeled by DL in section 3 and further discussed in section 4. 

To summarize, in this review we follow a tradition using a word sign for an arbitrary, amodal, static, unmotivated notation (unmotivated means unemotional, in particular). We use a word symbol for the PSS and DL processes-simulators, these are dynamic processes, connecting unconscious to conscious; they are motivationally loaded with emotions. As discussed in section 2, DL processes are motivated toward increasing knowledge, and they are loaded with knowledge-related emotions, even in absence of any other motivation and emotion. These knowledge-related emotions are called aesthetic emotions since Kant. They are foundations of higher cognitive abilities, including abilities for the beautiful, sublime, and they are related to musical emotions. More detailed discussions can be found in [25,49,51,52,53,54,55,57,66,77,78,91,92,93,94].

DL mathematical models (in section 3) use mathematical notations, which could be taken for amodal symbols. Such an interpretation would be erroneous. Meanings and interpretations of mathematical notations in a model depends not on the appearance, but on what is modeled. Let us repeat, any mathematical notation taken out of the modeling context, is a notation, a static sign. In DL model-processes these signs are used to designate neuronal signals, dynamic entities evolving “from vague to crisp” and mathematically modeling processes of PSS simulators-symbols. Upon convergence of DL-PSS simulator processes, the results are approximately static entities, approximately logical, less grounded and more amodal. 

DL models both, grounded, dynamic symbol-processes, overcoming combinatorial complexity and amodal static symbols, which are governed by classical logic and in the past have led to combinatorial complexity. DL operates on a non-logical type of PSS representations, which are vague combinations of lower-level representations. These lower-level representations are not necessarily complete images or events in their entirety, but could include bits and pieces of various sensor-motor modalities, memory states, as well as vague dynamic states from concurrently running simulators—DL processes of the on-going perception-cognition. (In section 3, for simplicity of presentation, we assumed that the lower-level object-simulators have already run their course and reached static states; however, the same mathematical formalism can model simulators running in parallel on multiple hierarchical levels.) The mind-brain is not a strict hierarchy, the same-level and higher-level representations could be involved along with lower levels. DL models processes-simulators, which operate on PSS representations. These representations are vague and incomplete, and DL processes are assembling and concretizing these representations. As described in several references by Barsalou, bits and pieces from which these representations are assembled could include mental imagery as well as other components, including multiple sensor, motor, and emotional modalities; these bits and pieces are mostly inaccessible to consciousness during the process dynamics.

DL also explains how logic and ability to operate amodal symbols originate in the mind from illogical operations of PSS: mental states approximating amodal symbols and classical logic appear as the end of the DL process-simulators. At this moment they become conscious static representations and loose that component of their emotional-motivational modality, which is associated with the need for knowledge (to qualify as amodal, these mental states should have no sources of modality, including emotional modality). The developed DL formalization of PSS, therefore suggests using a word signs for amodal mental states as well as for amodal static logical constructs outside of the mind, including mathematical notations; and to reserve symbols for perceptually grounded motivational cognitive processes in the mind-brain. Memory states, to the extent they are static entities, are signs in this terminology. Logical statements and mathematical signs are perceived and cognized due to PSS simulator symbol-processes and become signs after being understood. Perceptual symbols, through simulator processes, tie together static and dynamic states in the mind. Dynamic states are mostly outside of consciousness, while static states might be available to consciousness.

## 4. Abstract Concepts, Language, and the Mind Hierarchy

Here we discuss DL as a general model of interacting bottom-up and top-down signals throughout the hierarchy-heterarchy of the mind-brain, including abstract concepts. The DL mathematical analysis suggests that modeling the process of learning abstract concepts has to go beyond PSS analysis. In particular, we discuss the role of language in learning abstract concepts [73,93,95,96,97,98,99,100,101,102] and connect it to the PSS mechanisms. The mind-brain is not a strict hierarchy, interactions across levels are present and this is sometimes addressed as heterarchy [41]. To simplify discussion we would use a term hierarchy.

Section 2 discussed assembling situation representations from object representations. This addresses interaction between top-down and bottom-up signals in two adjacent levels of the mind hierarchy. The mathematical description presented in section 2 (and the Appendix) addresses top-down and bottom-up signals and representations without explicit emphasis on their referring to objects or situations. Accordingly, we would emphasize here that the mathematical formulation underlining section 2 analysis equally addresses interaction between any two adjacent levels in the entire hierarchy of the mind-brain, including high-level abstract concepts. DL overcomes the ubiquitous problem of CC, the presented DL mathematics is practically computable in a machine or mind. However, another fundamental aspect, grounding, remains questionable and is addressed below. 

In the PSS formulation Barsalou assumed that higher level abstract concepts remain grounded since they are based on lower level grounded concepts, and down the hierarchy to perceptions directly grounded in sensory-motor signals. The DL modeling suggests that this aspect of the PSS theory has to be revisited for the following two reasons. First, each higher level is vaguer than a lower level. Several levels on top of each other would result in representations too vaguely related to sensory-motor signals to be grounded in them with any reliability. Second, the section 2 example is impressive in its numerical complexity, which significantly exceeds anything that has been computationally demonstrated previously. We would like to emphasize again that this is mostly due to overcoming difficulty of CC. Still statistically, learning of situations was based on these situations being present among the data with statistically sufficient information to distinguish them among each other and from noise. In real life however, human learn complex abstract concepts, such as “state”, “law”, “rationality”, and many other abstract concepts, without statistically sufficient information been experienced (we return to this statement later and discuss its various aspects and a need for experimental verification). Now we would like to emphasize the role of language in learning abstract concepts. 

Language is learned at all levels of the hierarchy of the mind-brain and cultural knowledge from surrounding language. Experience of talking with other people operates in significant way with “ready made” language concepts. This makes it possible for kids to talk about much of cultural contents by the age of five or seven. At this age kids can talk about many abstract ideas, which they cannot yet adequately use in real life. This suggests that language concepts and cognitive concepts are different. Language concepts are grounded in surrounding language at all hierarchical levels. But learning corresponding cognitive concepts grounded in life experience takes an entire lifetime. Learning language, like learning cognition is driven by an inborn drive, the language instinct [103] (LI). Mathematically it can be modeled by DL [22,23,74,104]. Linguists consider words to be learned by memorizing them. Learning meaningful phrases and syntax is similar to learning situations and relations among objects in section 2. Morphology is not unlike object composition. 

## 5. The Dual Model: Joint Language and Cognition

**Figure 7:**
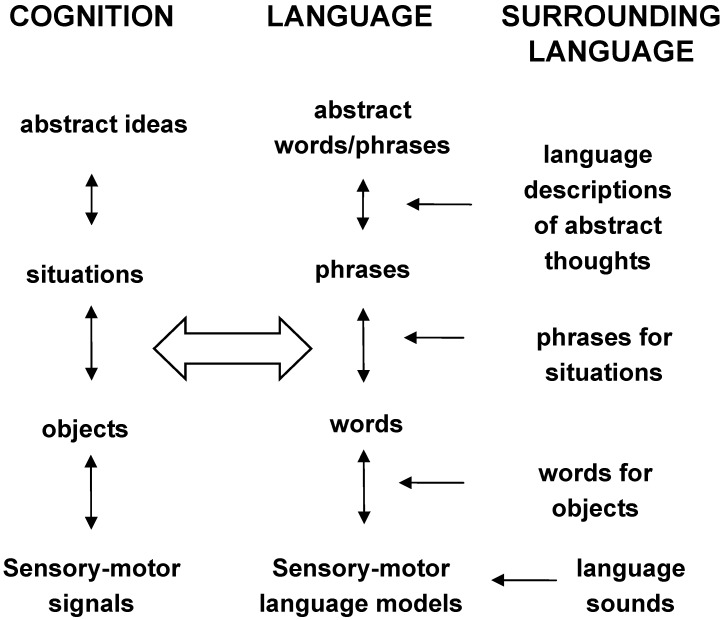
The dual-model architecture, modeling interaction of language and cognition. Learning of cognition is grounded in experience and guided by language. Learning of language is grounded in the surrounding language at all hierarchical levels.

A fundamental difference of LI from KI is that KI drives matching cognitive mental representations to objects and events in the world. LI drives matching language mental representations to patterns in the surrounding language. In other words this difference between language and cognition is called grounding [29,37,91,93,99]. Language is grounded in direct experience (of talking, reading) at all levels of the hierarchy, whereas cognition is grounded in direct perceptions only at the bottom of the hierarchy. Abstract concepts at every level are a tiny part of all possible combinations of bottom-up signals coming from the lower level. Useful combinations cannot be learned from life experience alone. Higher abstract levels of cognition are grounded in both language and experience. The detailed theory of interaction between cognition and language is considered in [29,48,94,96,105]. The main mechanism of interaction between language and cognition is the dual model, which suggests that every mental model-representation has two neurally connected parts, language model and cognitive model. Language models are learned by simulator processes, similar to PSS simulators, however, “perception” in case of language refers to perception of language facts. Through neural connections between the two parts of each model, the early acquired abstract language models guide the development of abstract cognitive models in correspondence with personal experience and cultural knowledge stored in language. The dual model leads to the dual hierarchy illustrated in Figure 7.

DL described in previous sections has overcome CC of learning situations and phrases. The simulation examples illustrated fast convergence. However from the analysis in the previous section it follows that human level cognition requires joint learning of language and cognition. This requires an architecture capable of learning two parallel hierarchies of Figure 7. This architecture models individual learning from a surrounding language with joint learning of cognition, as well as emergence of language and cognition in biological and cultural evolution. 

This dual-model suggests that the connection between language and cognitive models is inborn. In a newborn mind both types of models are vague mostly empty placeholders for future cognitive and language contents. An image, say of a chair, and the sound “chair” do not exist in a newborn mind. But the neural connections between the two types of models are inborn. This solves a problem of “association”: how words are associated with correct objects. A currently prevailing assumption that correct associations are learned from experience is untenable. The number of possible associations between words and objects (and abstract concepts) is combinatorially large. Because of this CC, no amount of experience would be sufficient for this learning. 

Instead, the brain-mind first learns language models from surrounding language; surrounding language contains language models “ready-made” and no real-life experience (beyond language) is needed; this language learning proceeds fast (as described by DL) from vague inborn contents to crisp knowledge of language by the age of about 5. Second, beginning with vague contents of cognitive model, vague associations with language models, and vague associations with various experiences, the brain-mind learns contents of cognitive models according to language and according to experience. So that cognitive contents become crisp (more or less) and always remain associated with the appropriate language models. 

The dual model is mathematically described in the Appendix. It solves several puzzles of learning language and cognition, which have remained unanswered ever since philosophy (since Locke 1690) and psychology attempted to explain them. These puzzles include: How does every child, even without schooling, learn correct correspondence between objects and words among zillions of possible correspondences? Why do children learn a language by 5 years of age and can talk about the entire cultural contents, but cannot act like adults? What exactly do children miss in terms of cognitive mechanisms? How do words get connected with objects and events? Why can animals not speak similarly to humans? How do language and cognition interact in thinking? Do we use words to label completed thoughts for communication? Or do we think with words? 

The dual model architecture, Figure 7, implies neural connections between cognitive and language representations. These connections are likely to be of ancient origins due to the mirror neuron system, MNS [106]. MNS implies neural connections between perceptual-cognitive brain areas, oro-laryngeal movement control, and the brain area, which in humans became Broca’s area fundamentally involved in language [107]. It is therefore considered a precursor to human language. M. Arbib [108] called these neural mechanisms “language prewired brain”.

Language models become less vague and more specific much faster than the corresponding cognitive models for the reason that they are acquired ready-made from the surrounding language. This is especially true about the contents of abstract models, which cannot be directly perceived by the senses, such as “law”, “abstractness”, “rationality”, *etc.* This explains how it is possible that kids by the age of five can talk about most contents of the surrounding culture, but cannot function like adults: cognitive models remain vague and gradually acquire more concrete contents throughout life. According to the dual-model hypothesis, this is an important aspect of the mechanism of what is colloquially called the “acquiring experience”. It would be important in future research first, to identify the detailed neural mechanisms of models, second, the neural mechanism of connections between language and cognitive models, and third, to correlate the suggested mechanism (of cognitive models becoming crisper) with the currently known maturation mechanism of myelination, reaching into adulthood. Here we emphasize what could the reason be for significant differences in speeds of learning language and cognitive models.

This dual-model hypothesis also suggests that the inborn neural connection between cognitive brain modules and language brain modules (evolving over thousands or millions of years of evolution) is fundamental to setting humans on an evolutionary path separating us from the animal kingdom. Eventually it led to rewiring emotional connections in the brain controlling voice, and evolution of the mental hierarchy.

Human learning of cognitive models continues through the lifetime and is guided by language models. Here we would like to reconsider the experiment with closed eyes described previously. It is virtually impossible to remember imagined perceptions when eyes are opened. Similarly, language plays a role of eyes for abstract thoughts. On one hand, abstract thoughts are only possible due to language, on the other, language “blinds” our mind to vagueness of abstract thoughts. Whenever one can talk about an abstract topic, he (or she) might think that the thought is clear and conscious in his (or her) mind. But the above discussion suggests that we are conscious about the language models of the dual hierarchy. The cognitive models in most cases may remain vague and unconscious. The higher up in the hierarchy the vaguer are the contents of abstract thoughts, while due to crispness of language models we may remain convinced that these are clear conscious thoughts.

The dual model further suggests a mechanism for what became known as basic human irrationality. The discovery of this was initiated in works of Tversky and Kahneman [109,110]; leading to a 2002 Nobel Prize. Language is significantly crisp in the human brain, while cognition might be vague. Using the KI mechanisms to arrive at rational decisions (to make cognitive models crisp) requires a special effort and training. Language accumulates millennial cultural wisdom and it might be to one’s advantage to rely on heuristics formulated in language. This suggestion is a scientific hypothesis that can be and should be verified experimentally. In this future verification it is necessary to carefully consider the role of emotions. It was suggested that irrational heuristic decision making *vs.* KI-deliberate analysis activates the amygdala stronger than the cortex [111]. So that emotions may play a larger role in irrational decision making. Two words of caution are due. First, emotional decision making could be perfectly rational and not necessarily related preferentially to amygdala. Second, these rational emotions might be different from specific language emotions considered in the next section.

## 6. Language Emotionality, or Emotional Sapir-Whorf Hypothesis

The KI drives the human brain to develop more specific, concrete and conscious cognitive models by accumulating experience throughout life in correspondence with the language models. For this process to remain active, brains have to maintain “motivation” to do it. This motivation is not automatic. We have suggested the hypothesis that there are specific emotions related to language [112,113]. The origin of language required freeing vocalization from uncontrolled emotional influences. Initial undifferentiated unity of emotional, conceptual, and behavioral-(including voicing) mechanisms had to separate, to differentiate into partially independent systems. Voicing separated from involuntary emotional control due to a separate emotional center in the cortex, which controls the larynx muscles, and which is partially under volitional control [79,114,115,116]. Evolution of this volitional emotional mechanism possibly paralleled the evolution of language computational mechanisms. In contemporary languages the conceptual and emotional mechanisms are significantly differentiated, as compared to animal vocalizations. The languages evolved toward conceptual contents, while their emotional contents were reduced. 

Language and voice started separating from ancient emotional centers possibly millions of years ago. Nevertheless, emotions are present in contemporary languages [106]. Emotionality in language is expressed in language sounds, what linguists call prosody or melody of speech. Emotions in language sounds may affect ancient emotional centers of the brain. This ability of human voice to affect us emotionally is most pronounced in songs. Songs and music, however, is a separate topic [117,118] addressed later.

Everyday speech is low in emotions, unless affectivity is specifically intended. We may not notice emotionality of everyday “non-affective” speech. Nevertheless, “the right level” of emotionality is crucial for developing the cognitive parts of models. If language parts of the models were always highly emotional, any discourse would immediately resort to conflict and there would be no room for language development (as among primates). If the language parts of models were always non-emotional, there would be no motivational force to engage into conversations, and to develop cognitive models. The motivation for developing higher cognitive models would possibly be reduced. Lower cognitive models, say for object perception, would be developed because they are imperative for survival and because they can be developed independently from language, based only on direct sensory perceptions, like in animals. But models of situations and higher cognition (as discussed) are developed based on language models. This requires emotional connections between cognitive and language models. 

Primordial fused language-cognition-emotional models have differentiated long ago. The involuntary connections between voice-emotion-cognition have dissolved with the emergence of language. They have been replaced with habitual connections. The sounds of all languages have changed and, it seems, sound-emotion-meaning connections in languages should have been severed. Nevertheless, if the sounds of a language change slowly, connections between sounds and meanings persist and consequently the emotion-meaning connections persist. This persistence is a foundation of meanings because meanings imply motivations. If the sounds of a language change too fast, the cognitive models are severed from motivations, and meanings disappear. If the sounds change too slowly the meanings are fixed emotionally to the old ways, and culture stagnates.

This statement is a controversial issue, and indeed, it may sound puzzling. Does culture not direct language changes or is the language the driving force of cultural evolution? Theoretical considerations suggest no neural or mathematical mechanism for culture directing evolution of language through generations; just the opposite, most cultural contents are transmitted through language. Cognitive models contain cultural meanings separate from language [99], but transmission of cognitive models from generation to generation is mostly facilitated by language. Cultural habits and visual arts can preserve and transfer meanings, but they contain a minor part of cultural wisdom and meanings comparative to those transmitted through the language. Language models are major containers of cultural knowledge shared among individual minds and collective culture. Existing experimental evidence supports this view [119,120].

The arguments in the previous two paragraphs suggest that an important step toward the understanding of cultural evolution is to identify the mechanisms determining the changes of the language sounds. As discussed below, changes in language sounds are controlled by grammar. In inflectional languages, affixes, endings, and other inflectional devices are fused with sounds of word roots. Pronunciation-sounds of affixes are controlled by few rules, which persist over thousands of words. These few rules are manifest in every phrase. Therefore every child learns to pronounce them correctly, even if the child does not know which case or inclination to use in which sentence. Positions of vocal tract and mouth muscles for pronunciation of affixes (*etc.*) are fixed throughout a population and are conserved throughout generations. Correspondingly, the pronunciation of whole words cannot vary too much, and language sound changes slowly. Inflections therefore play a role of “tail that wags the dog” as they anchor language sounds and preserve meanings. This, we suggest is what Humboldt [121] meant by “firmness” of inflectional languages. When inflections disappear, this anchor is no more and nothing prevents the sounds of language to become fluid and change with every generation. 

This has happened with the English language after transition from Middle English to Modern English [122], most of inflections have disappeared, a great vowel shift occurred, and the sound of the language started changing within each generation, with this process continuing today. English evolved into a powerful tool of cognition unencumbered by excessive emotionality. The English language spreads democracy, science, and technology around the world. This has been made possible by conceptual differentiation empowered by language, which overtook emotional synthesis. But the loss of synthesis has also lead to ambiguity of meanings and values. Current English language cultures face internal crises, uncertainty about meanings and purposes. Many people cannot cope with the diversity of life. Future research in psycholinguistics, anthropology, history, historical and comparative linguistics, and cultural studies will examine interactions between languages and cultures. Initial experimental evidence suggests emotional differences among languages consistent with our hypothesis [113,114,123].

The opposite case is when emotional involvement is too strong. In this case learning does not take place because old knowledge is perceived as too valuable, and no change is possible. This case might be characteristic of “too strongly” inflected languages, in which sound changes “too slowly” and emotions are connected to meanings “too strongly”. A brief look at cultures and languages certainly points to many examples of this case: highly inflected languages and correspondingly “traditional” stagnating cultures. Preliminary analysis indicates some languages with strong fusional inflexitons as examples of highly emotional language with stagnating cultural evolution [124,125]. The other side of strong language emotionality is a strong feel of identity and purpose. This corresponds to Humboldtian analysis. The *integrated* dual model assumes “moderate” emotional connection between language and cognitive models, which fosters the integration and does not impede it. Humboldt suggested that this relationship is characteristic of inflectional languages (such as Indo-European), inflection provided “the true inner firmness for the word with regard to the intellect and the ear” (today we would say “concepts and emotions”). The integrated dual model leads to interaction between language and cognition and to accumulation of knowledge. This accumulation, however, does not proceed smoothly; it leads to instabilities and oscillations, possibly to cultural calamities; this characterizes significant part of European history from the fall of Roman Empire to recent times. 

Much of contemporary world is “too flat” for an assumption of a single language and culture, existing without outside influences. In case of mutual influences of cultures, when cultures with strongly emotional and weekly emotional languages intereact, the opposite tendencies of these cultures may counterbalance each other, so that both evolve faster and more smoothly [67,70,91,106,109,126].

The neural mechanisms of grammar, language sound, related emotions-motivations, and meanings hold a key to connecting the neural mechanisms in the individual brains to the evolution of cultures. Studying them experimentally is a challenge for future research. Necessary experimental methodologies are at hand; they just should be applied to these issues, and several research groups pursue these experiments.

## 7. Cognitive Function of Music

When discussing KI previously we emphasized its function of maximizing a similarity between bottom up and top down signals. However, a single measure of similarity is too narrow for measuring human knowledge. Every two concepts-representations contradict each other to some extent (otherwise a single concept will be sufficient). 

Contradictions between different aspects of knowledge are difficult to tolerate. These contradictions are called cognitive dissonances, and this is a well studied area of psychology [127]. Ancient Greeks new that people tend to resolve the dissonances by devaluing a conflicting cognition. In the Aesop’s fable The Fox and the Grapes a fox sees high-hanging grapes. A desire to eat grapes and inability to reach them are in conflict. The fox overcomes this cognitive dissonance by deciding that the grapes are sour and not worth eating. Since the 1950s cognitive dissonances became a wide and well studied area of psychology. It is known that tolerating cognitive dissonances is difficult, and people often make irrational decisions to avoid them [104]. In 2002 this research was awarded Nobel Prize in economics, emphasizing the importance of this field of research. 

Perlovsky [56,88,89,111,112,128,129,130] emphasized that the evolution of language led to relatively fast cultural evolution of multiple mutually contradictory concepts (any different concept must be contradictory to some extent; otherwise one concept would be sufficient). This created cognitive dissonances, which consequently could lead to devaluing knowledge [43]. If cognitive dissonance could not be mitigated, our progenitors would devalue knowledge, and human language, knowledge, and culture would not evolve. The above references hypothesized that the fundamental function of music in cognition was to serve precisely this function, to mitigate cognitive dissonances. Other testable predictions, address the number of emotions of cognitive dissonances and musical emotions. Most studies of emotions address “basic emotions”, which are named by emotional words and indicate satisfaction of bodily instincts [131]. Since the number of cognitive dissonances is combinatorially large, it is predicted that the number of musical emotions is also combinatorially large (practically infinite).

Debates about the function and origin of music have a long history. Aristotle [35] listed the power of music among the unsolved problems: “How music being just sounds reminds states of soul?” Kant [50], who so brilliantly explained the epistemology of the beautiful and the sublime, could not explain music. According to Darwin [132], the human musical faculty “must be ranked amongst the most mysterious with which (man) is endowed” because “music is a human cultural universal that appears to serve no obvious adaptive purpose” [133]. While some scientists argue that music itself plays no adaptive role in human evolution, others suggest that music clearly has an evolutionary role, and point to music’s universality. In 2008, Nature published a series of essays on music authored by authorities in evolutionary psychology [134]. The authors agreed that music is a cross-cultural universal, still “none... has yet been able to answer the fundamental question: why does music have such power over us?”, “We might start by accepting that it is fruitless to try to define ‘music’” [135]. 

In [136] an experimental confirmation has been obtained that music can reduce cognitive dissonances. These findings are tentatively supported by known physiology of brain mechanisms. Previous research demonstrated involvement of the anterior cingulate gyrus in creating cognitive dissonances [137]. At the same time, it is known that listening music decreases activity of the ventral medial prefrontal cortex as well as the limbic system, making listening more pleasurable, so that activation of the anterior cingulate gyrus is decreased [138].

## 8. Experimental Evidence

Bar *et al.* [4] demonstrated in neuroimaging experiments that visual perception proceeds according to DL simulating crisp perceptions from initial vague representations (In this reference authors use terminology of “low spatial frequency” for what we call vague). Experimental procedures in this reference used functional Magnetic Resonance Imaging (fMRI) to obtain high-spatial resolution of processes in the brain, which they combined with magneto-encephalography (MEG), measurements of the magnetic field next to the head, which provided high temporal resolution of the brain activity. Combining these two techniques the experimenters were able to receive high resolution of cognitive processes in space and time. Bar *et al.* [4] concentrated on three brain areas: early visual cortex, object recognition area (fusiform gyrus), and object-information semantic processing area (OFC). They demonstrated that OFC is activated 130 ms after the visual cortex, but 50 ms before object recognition area. Their conclusion has been that OFC represents the cortical source of top-down facilitation in visual object recognition. This top-down facilitation is unconscious. They demonstrated that the imagined image generated by top-down signals facilitated from OFC to cortex is vague (the authors in this publication refer to low spatial-frequency content images), confirming the essential mechanism of DL. Conscious perception of an object occurs when vague projections become crisp and match the crisp and clear image from the retina, and an object recognition area is activated. 

The brain continuously extracts rudimentary information from early sensory data and simulates predictions, which facilitate perception and cognition in the relevant context by pre-sensitizing relevant representations. This includes predictions of complex information, such as situations and social interactions [139,140]. Predictions are initiated by gist information rapidly extracted from sensory data. At the “lower”-object level this gist information is a vague image of an object (low spatial frequency, Bar *et al.* [4]). At higher levels “the representation of gist information is yet to be defined”. The described model defines this higher-level gist information as vague collections of vague objects, with relevant objects for a specific situation having just slightly higher probabilities than irrelevant ones. The developed model is also consistent with the hypothesis in [43] that perception and cognition at higher levels relies on mental simulations. Mathematical predictions in this paper suggest specific properties of these higher-level simulators (initial top-down representations are vague in terms of their associations with bottom-up signals), which could be verified experimentally. The DL process from vague to crisp and from unconscious to conscious is a fundamental mechanism of the brain.

Existence of specific emotion involved with knowledge, aesthetic emotions, and therefore of KI has been demonstrated experimentally [46]. 

Different languages have different strengths of emotional connections between sounds and cognitive meanings of words. These different emotionalities of languages have been experimentally confirmed in [113,114,117]. Franklin *et al.* [141] demonstrated that the direction of influence is indeed from language to brain (not the opposite) and hence from language to culture. Empirical evidence supports predicted relations between language flectivity and cultural evolution.

## 9. Future Research

Future research will address the DL mathematical description of PSS throughout the mind hierarchy; from features and objects “below situations” in the hierarchy to abstract models and simulators at higher levels “above situations”. Modeling across the mind modalities will be addressed including diverse modalities, symbolic functions, conceptual combinations and predication. Modeling features and objects would have to account for suggestions that perception of features is partly inborn [21]; this development therefore might require new experimental data on which feature aspects are inborn [142]. The developed DL formalization of PSS corresponds to observations in [143] and it will be used for generating more detailed experimentally verifiable predictions. A number of predictions have been made in this review, including influence of language on cognition. 

The proposed theory provides solutions to classical problems of conceptual relations, binding, and recursion. Binding is a mechanism connecting events into meaningful “whole” (or larger-scale events). The DL model developed here specifies two types of binding mechanisms “flat” and “hierarchical”, and suggests which mechanisms are likely to be used for various relations. Our model also suggests existence of binding mechanisms conditioned by culture and language. Recursion has been postulated to be a fundamental mechanism in cognition and language [144], however, that reference has not proposed specific mechanisms how recursion creates representations, nor how it maps representations into the sensory-motor or conceptual-intentional interfaces. In our opinion this is an erroneous assumption, and the error is similar to mistaking logical signs for symbol-processes (recursion is an important logical operation). According to the developed theory recursion is not a fundamental mechanism, instead the hierarchy is a mechanism of recursion. Successive hierarchical levels accomplish recursive cognitive and linguistic functions. Again, these predictions can be experimentally tested.

Experimental research [4,43] can address specific properties of higher-level simulators predicted here. Among these is a prediction that early predictive stages of situation simulations are vague. Whereas vague predictions of objects resemble low-spatial frequency of object imagery, “the representation of gist information on higher levels of analysis is yet to be defined” [43]. According to the developed model, vague predictions of situations should contain many less-relevant (and likely vague) objects with lower probabilities. Since the mathematical model proposed here is applicable to higher levels (“above” object-situations), this hypothesis should be relevant to the nature of information of higher-level gist: initial representation of abstract concepts are vague in terms of associations with constituent bottom-up signals (these associations are not exact, but vague; probabilistically, they are not close to zeroes and ones). 

The present model addresses another topic discussed in [43], “how the brain integrates and holds simultaneously information from multiple points in time”. Two different mechanisms are likely to be involved: first, explicit incorporation of time into models (so that model parameters-probabilities depend on time), and second, categorized temporal relations, such as “before”, “after” are included as any other relations-objects into models. A joint mathematical-experimental approach might be fruitful in this area.

Future research will address interaction between language and cognition. Language is acquired from surrounding language, rather than from direct experience in the world; language therefore is closer aligned with amodal symbols than with perceptual symbols. Kids at 5 years of age can talk about much of cultural content of the surrounding language, including highly abstract contents; yet, clearly kids do not have necessary experience to understand highly abstract concepts, as perceptual symbols, and to relate them to the world. According to the developed theory, higher abstract concepts could be stronger grounded in language than in perception; not only kids, but also adults may operate with abstract concepts as with amodal symbols, and therefore have limited understanding grounded in experience of how abstract concepts relate to the world. It follows that higher-level concepts may be less grounded in perception and experience than in language. A brain implementation of dual model through nonlinear dynamics discussed in [145] The developed theory suggests several testable hypotheses: the dual model, postulating separate cognitive and language mental representations, neural connections between cognitive and language mental representations, language mental representations guiding acquisition of cognitive representations in ontological development, abstract concepts being more grounded in language than in experience; and this shift from grounding in perception and experience to grounding in language progresses up the hierarchy of abstractness; while grounding in perception and experience increases with age. These make a fruitful field for future experimental research.

The suggested model of connections between language and cognition bears on language evolution, and future research should address theoretical and experimental tests of this connection between evolution of languages, cognition, and cultures [37,90,92,100,106,146].

The role of emotions in perception was addressed in [147]. There are several mechanisms of emotions and future research should extend this paper formalism to more detailed modeling of emotions and their role in cognition. The current review emphasizes functions of aesthetic emotions specific to knowledge and related to KI. Future research would explore roles of emotions in language-cognition interaction [106] and in symbol grounding. Discussions in this review predict that aesthetic emotions in cognitive dissonances are closely related to musical emotions. Whereas basic emotions that have been mostly studied by psychologists are few in number, emotions of cognitive dissonances could be combinatorially large corresponding to the number of combinations of various concepts (practically infinite). Similarly, there could be a practically infinite number of musical emotions [88,89,111,121,128,148].

To understand brain-mind mechanisms from perception to the highest cognitive and emotional abilities it is necessary to understand interactions between conscious and unconscious brain states.

## 10. Conclusion

This review has summarized conscious and unconscious brain mechanisms, including cognition, emotions, language and interaction between language and cognition. The fundamental mechanisms of cognition include interactions between bottom-up and top-down signals. The difficulties of modeling these interactions since the 1960s are related to combinatorial complexity (CC), and fundamental reasons for CC are related to the Gödel’s difficulties of logic, a most fundamental mathematical result of the 20th century. The review discusses that conscious states in the mind-brain are approximately logical, whereas an overwhelming number of non-logical states and processes in the brain are inaccessible to consciousness. This is the reason for many scientists still “believing” in logic as fundamental to the brain. CC difficulty is overcome in the brains by processes “from vague-unconscious to crisp-conscious” (representations, plans, models, concepts). These processes are modeled by dynamic logic, a process from vague and unconscious representations toward crisp and conscious thoughts. We discuss experimental proofs and relate dynamic logic to simulators of the perceptual symbol system. “From vague-unconscious to crisp-conscious” is a fundamental mechanism of the brain. In addition to perception and cognition, it explains interactions between cognition and language. Language is mostly conscious, whereas cognition is only rarely so; this clarifies much about the mind-brain that might have seemed mysterious. All of the above involve emotions of a special kind, aesthetic emotions related to knowledge and to cognitive dissonances. Cognition-language-emotional mechanisms operate throughout the hierarchy of the mind and create all higher mental abilities. The review discusses cognitive functions of the beautiful, sublime, music.

## Appendix

Bottom-up signals {**X**(*n*)} in this simplified discussion, is a field of neuronal synapse activations in visual cortex. Here and below curve brackets {…} denote multiple signals, a field. Index *n* = 1, … *N*, enumerates neurons and **X**(*n*) are the activation levels. Sources of top-down signals are representations or concept-models {**M***m*(*n*)} indexed by *m* = 1, … *M*. Each model m **M***m*(*n*) projects a set of priming, top-down signals, representing the bottom-up signals **X**(*n*) expected from a particular object, *m*. Models depend on parameters {**S***m*}, **M***m*(**S***m*,*n*). Parameters characterize object position, angles, lightings, *etc.* (In case of learning situations considered in section 3, parameters characterize objects and relations making up a situation.) To summarize this highly simplified description of a visual system, *n* enumerates the visual cortex neurons, **X**(*n*) are the “bottom-up” activation levels of these neurons coming from the retina, and **M***m*(*n*) are the “top-down” activation levels (priming) of the visual cortex neurons. The learning-perception process “matches” these top-down and bottom-up activations by selecting “best” models and their parameters and the corresponding sets of bottom-up signals.

Let us concentrate on defining a mathematical measure of the “best” fit between bottom-up and top-down signals. It is constructed in such a way that any of object-models can be recognized. Correspondingly, a similarity measure is designed so that it treats each object-model as a potential alternative for each subset of signals [25,30],

(A1)

Here, l(**X**(*n*)|**M***m*(*n*)) (or simply l(*n*|*m*)) is called a conditional similarity between one signal **X**(*n*) and one model **M***m*(*n*). Parameters r(*m*) are proportional to the number of objects described by the model *m*. Expression (A1) accounts for all combinations of signals and models in the following way. Sum ∑ ensures that any of the object-models can be considered (by the mind) as a source of signal **X**(*n*). Product ∏ ensures that all signals have a chance to be considered (even if one signal is not considered, the entire product is zero, and similarity *L* is 0; so for good similarity all signals have to be accounted for. This does not assume exorbitant amount of attention to each minute detail: among models there is a vague simple model for “everything else”). In a simple case, when all objects are perfectly recognized and separated from each other, there is just one object-model corresponding to each signal (other l(*n*|*m*) = 0). In this simple case expression (A1) contains just 1 item, a product of all non-zero l(*n*|*m*). In the general case, before objects are recognized, *L* contains a large number of combinations of models and signals; a product over N signals is taken of the sums over M models; this results in a total of MN items; this huge number is the cause for the combinatorial complexity discussed previously.

The DL learning process consists in estimating model parameters **S***m* and associating subsets of signals with concepts by maximizing the similarity (A1). Although (A1) contains combinatorially many items, DL maximizes it without combinatorial complexity [25,27,30,56,71,72]. First, fuzzy association variables f(*m*|*n*) are defined,

(A2)

These variables give a measure of correspondence between signal **X**(*n*) and model **M***m* relative to all other models, *m*′. They are defined similarly to the a posteriori Bayes probabilities, they range between 0 and 1, and as a result of learning they converge to the probabilities under certain conditions.

DL process is defined by the following set of differential equations,

(A3)
